# Insights into the Factors Controlling the Origin of Activation Barriers in the [2 + 2] Cycloaddition Reactions of Heavy Imine-like Molecules Featuring a Ge=Group 15 Double Bond with Heterocumulenes

**DOI:** 10.3390/molecules30091905

**Published:** 2025-04-25

**Authors:** Zheng-Feng Zhang, Ming-Der Su

**Affiliations:** 1 Department of Applied Chemistry, National Chiayi University, Chiayi 60004, Taiwan; ftg17669@gmail.com; 2 Department of Medicinal and Applied Chemistry, Kaohsiung Medical University, Kaohsiung 80708, Taiwan

**Keywords:** [2 + 2] cycloaddition, heterocumulene, heavy imine, donor–acceptor interaction, electron-sharing

## Abstract

The [2 + 2] cycloaddition reactions of the heterocumulene (**N=C=N**) with the heavy imine-like molecule **Ge=G15-Rea** (G15 = Group 15 element) were examined using density functional theory (M06-2X-D3/def2-TZVP). The theoretical findings indicate that the doubly bonded Ge=G15 moiety in **Ge=G15-Rea** (L_1_L_2_Ge=G15L_3_) is characterized by electron-sharing bonding between the triplet L_1_L_2_Ge and triplet G15–L_3_ fragments. All five Ge=G15-based heavy imine analogues readily undergo [2 + 2] cycloaddition reactions with **N=C=N**. Energy decomposition analysis (EDA–NOCV) suggests that the [2 + 2] cycloaddition reaction between **Ge=G15-Rea** and **N=C=N** involves a donor–acceptor (singlet–singlet) interaction instead of an electron-sharing (triplet–triplet) interaction. Frontier molecular orbital (FMO) theory and the energy decomposition analysis–natural orbitals for chemical valence (EDA–NOCV) findings emphasize that the key bonding interaction involves the occupied p-π orbital of **Ge=G15-Rea** and the vacant p-π* orbital of **C=N=C**. Based on the activation strain model results, the activation barrier of the [2 + 2] cycloaddition reaction is predominantly controlled by the deformation energies of **Ge=G15-Rea** and **N=C=N**.

## 1. Introduction

Historically, the creation of heavier element–element multiple bonds was regarded as unfeasible, resulting in the “double-bond rule” [1,2]. This rule highlights challenges like diminished p-orbital overlap and increased Pauli repulsion, which hinder synthetic efforts. The discovery of Si=Si [3] and P=P [4] double bonds in the 1980s spurred a new era of research into heavier diatomic multiple bonds, resulting in the isolation of heavier organic analogs [5,6,7,8,9,10,11,12,13,14,15,16,17,18]. Unlike their lighter counterparts, these analogs exhibit unique differences in stability, bonding behavior, and chemical properties, attributed to the minimal s/p hybridization seen in heavier elements. Their distinguishing features have propelled them into a pivotal position in synthetic chemistry, driving the development of unique compounds and the optimization of catalytic methodologies [5,6,7,8,9,10,11,12,13,14,15,16,17,18,19,20,21,22].

Known for their bottle stability and widespread use, imines (R_2_C=NR′) are key organic compounds. Heavier analogs, on the other hand, exhibit significantly enhanced reactivity, as confirmed by experimental and theoretical studies [23,24,25,26,27,28,29,30]. This increased reactivity is a consequence of the greater polarity of their multiple bonds, resulting from weaker orbital overlap in heavier atoms [31,32,33,34,35]. Within this framework, germanimines featuring Ge=N double bonds have attracted considerable attention from researchers [36,37,38,39,40,41,42,43,44,45]. These compounds are considered heavier analogs of organic imines. The Ge=N bond, in contrast to the C=N bond, is significantly polarized due to poor p orbital overlap between germanium and nitrogen, resulting in high reactivity and synthetic difficulties [15,22]. Although challenging, the efforts of proficient synthetic chemists, along with the strategic use of bulky substituents, have made it possible to isolate and characterize heavier imine analogs containing a >Ge=N– skeleton [36,37,38,39,40,41,42,43,44,45]. Certain compounds additionally rely on stabilization by a Lewis acid or base [38,39,40,41]. While chemists have demonstrated significant success in synthesizing compounds containing >Ge=P– [6,8,46,47,48,49,50,51] and >Ge=As– [52,53] structures, it is surprising that >Ge=Sb– and >Ge=Bi– analogs, characterized by a tricoordinate germanium center and resembling organic imines (>C=N–), remain untouched in both experimental and computational research.

**Scheme 1:**
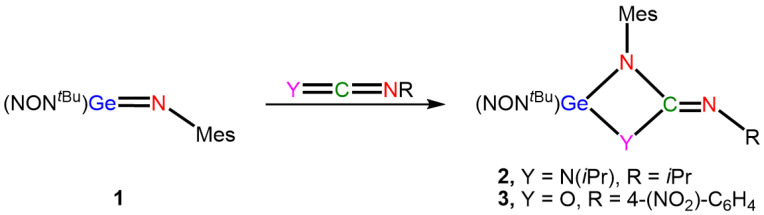
The [2 + 2] cycloaddition of heterocumulenes (**2** and **3**) with [(NON*^t^*^Bu^)Ge=N–Mes] (**1**, NON*^t^*^Bu^ = O(SiMe_2_N*t*Bu)_2_ and Mes = 2,4,6-Me_3_C_6_H_2_). See references [42,43].

A novel germanimine, [(NON*^t^*^Bu^)Ge=NMes] (**1**, NON*^t^*^Bu^ = O(SiMe_2_N*t*Bu)_2_ and Mes = 2,4,6-Me_3_C_6_H_2_), was recently reported by Fulton and co-workers [42,43]. This compound demonstrates metalloid-like characteristics, showing reactivity akin to transition metal–imido complexes. It undergoes [2 + 2] cycloadditions with heterocumulenes and protic sources, as well as [4 + 2] cycloadditions (metallo–Diels–Alder reactions) with activated dienes [38]. For example, **1** reacts with dicyclohexylcarbodiimide and 4-nitro-phenyl isocyanate to form germanium–guanidinate **2** and germanium ureate **3**, respectively. These compounds both feature germacycles with dianionic bidentate guanidinate and ureate ligands (Scheme 1) [42,43].

**Scheme 2:**
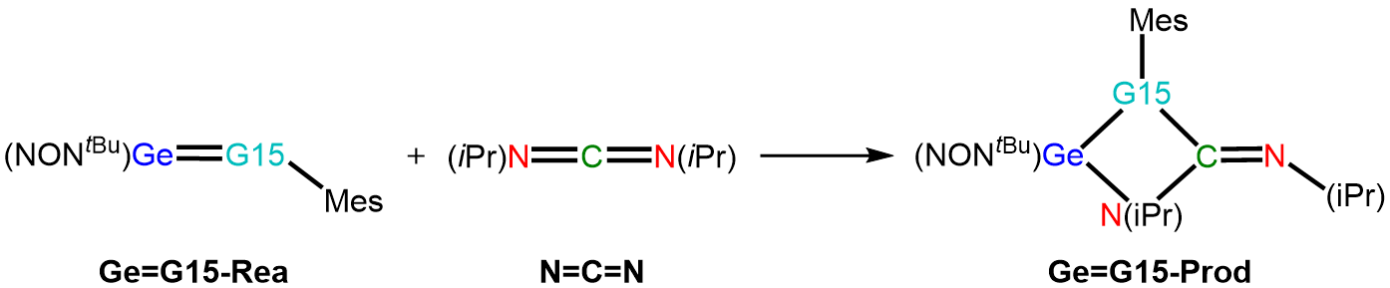
The [2 + 2] cycloaddition reaction model features ((NON*^t^*^Bu^)Ge=G15–Mes) (**Ge=G15-Rea**, NON*^t^*^Bu^ = O(SiMe_2_N*t*Bu)_2_) reacting with the doubly bonded heterocumulene **N=C=N**, also known as dicyclohexylcarbodiimide. Rea = reactant and Prod = product.

The fascinating experimental findings outlined above have sparked our interest and motivated us to delve deeper into this area of research. Although the reactions of germanimines, featuring a Ge=N double bond, with doubly bonded organic reagents have been extensively investigated by experimental chemists [54,55], their interactions with other doubly bonded species have been relatively overlooked, despite their potential importance in synthesizing and applying heavy analogues with a >Ge=G15– (G15 = Group 15 element) structure. Our objective is to examine how the replacement of the N atom in germanimines with other heavy Group 15 elements impacts the reactivity in [2 + 2] cycloaddition reactions. The primary focus of our inquiry is to understand the reactivity and the determinants of activation barriers in these reactions. Because they are limited by experimental constraints, theoretical computations now play a pivotal role in quickly predicting outcomes. We intend to apply density functional theory (DFT) and state-of-the-art methods to study the formation of [2 + 2] cycloaddition products between a heavy imine (>Ge=G15–) analog, composed of germanium and Group 15 element, and a heteroalkene, using the [2 + 2] reaction illustrated in Scheme 2 as a model. This effort seeks to facilitate the work of experimental chemists in optimizing synthesis and discovering novel applications.

## 2. Results and Discussion

### 2.1. The Nature of the Ge=G15 Bonding in Ge=G15-Rea

**Figure 1:**
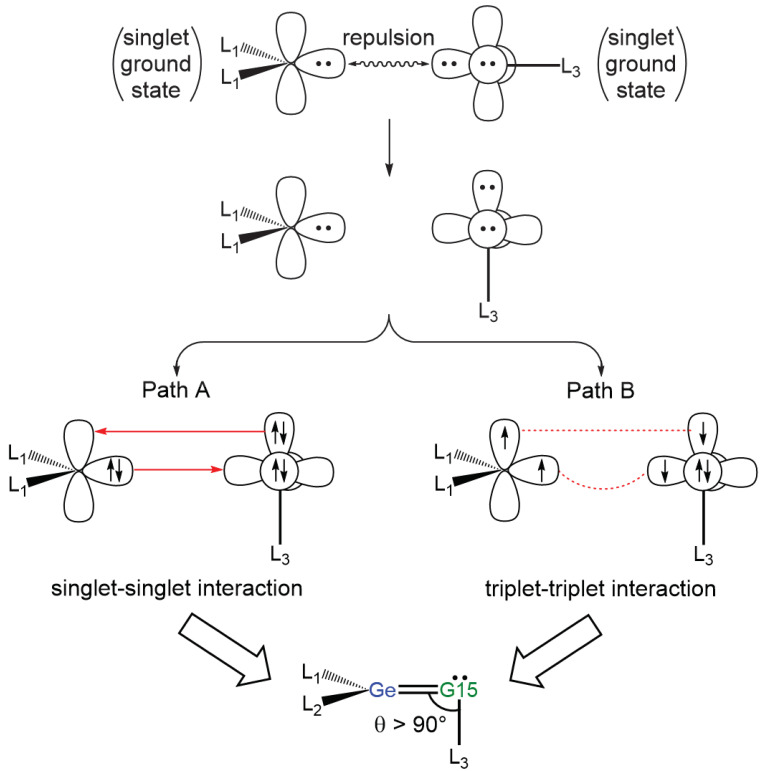
The valence bond bonding paths, path A and path B, for the doubly bonded **Ge=G15-Rea** ((NON*^t^*^Bu^)Ge=G15–Mes) molecule. Valence bond theory predicts that the bond angle ∠Ge–G15–L_3_ (θ) in **Ge=G15-Rea** should be greater than 90° due to steric interactions between the attached ligands. The arrows stand for donor-acceptor interaction.

To understand the chemical bonding of the Ge=G15 double bond, valence bond theory suggests dividing the (L_1_L_2_)Ge=G15L_3_ molecule into two units: (L_1_L_2_)Ge and G15−L_3_ [56]. In terms of spin, two possible interaction pathways can merge the moieties to form a double-bonded (L_1_L_2_)Ge=G15L_3_ molecule in the singlet ground state. The first, the “singlet–singlet interaction” or “donor–acceptor interaction”, is [(L_1_L_2_)Ge:]^1^ + [:G15–L_3_]^1^ → [(L_1_L_2_)Ge=G15L_3_]^1^ (path A), and the second, the “triplet–triplet interaction” or “electron-sharing interaction”, is [(L_1_L_2_)Ge:]^3^ + [:G15–L_3_]^3^ → [(L_1_L_2_)Ge=G15L_3_]^1^ (path B). The valence bond orbital interactions are illustrated in Figure 1. As presented in Figure 1, our theoretical study indicates that the bond angle ∠Ge–G15–L_3_ in (L_1_L_2_)Ge=G15–L_3_ assumes a bent geometry, likely greater than 90° due to steric hindrance from the ligands. Fulton et al. [42,43] corroborate this with X-ray data, showing a bond angle of 126.66° for ∠Ge–N–^Mes^C in **Ge=N-Rea**, which aligns with our theoretical findings.

Figure 1 illustrates that the pathway leading to the formation of (L_1_L_2_)Ge=G15–L_3_ is governed by the promotion energies of the units involved. Table S1 (Supporting Information) presents our M06-2X calculations, which establish that the (L_1_L_2_)Ge: ((L_1_L_2_) = NON*^t^*^Bu^) component has a singlet ground state, whereas the :G15–L_3_ unit (L_3_ = Mes) adopt a triplet ground state. The computed singlet–triplet energy gap, Δ*E*_ST_ (=*E*_triplet_ − *E*_singlet_), for the former is 42.7 kcal/mol, while for the latter (:G15–Mes; G15 = N, P, As, Sb, and Pb), it ranges from −31.0 to −28.6 kcal/mol.

The investigation continues with an in-depth analysis of the Ge=G15 bonding in doubly bonded, heavy imine-like **Ge=G15-Rea** molecules, employing EDA–NOCV [57,58,59,60,61,62,63,64,65,66]. As indicated in previous theoretical studies [67,68], Δ*E*_Orb_ (orbital interaction energy) serves as the key indicator of bonding characteristics, capturing the total change in orbital interactions between fragments. Fragments with the smallest energy variation during bond formation provide the clearest insight into their electronic structure. Table 1 illustrates our M06-2X analysis, which suggests that the absolute |Δ*E*_Orb_| value for the “triplet–triplet interaction” (81.3–251.8 kcal/mol) is lower than that of the “singlet–singlet interaction” (163.8–334.4 kcal/mol). This suggests that the “triplet–triplet interaction” is essential in characterizing the bonding nature of the Ge=G15 bond in heavy imine-like **Ge=G15-Rea** molecules. In brief, our theoretical analysis provides clear evidence that the “electron-sharing interaction” between the triplet L_1_L_2_Ge and triplet G15–L_3_ moieties plays a predominant role in the characterization of the Ge=G15 double bond in heavy imine analogs (**Ge=G15-Rea**).

### 2.2. The [2 + 2] Cycloaddition Reactions of Ge=G15-Rea with N=C=N Outlined in Equation (1)

**Figure 2:**
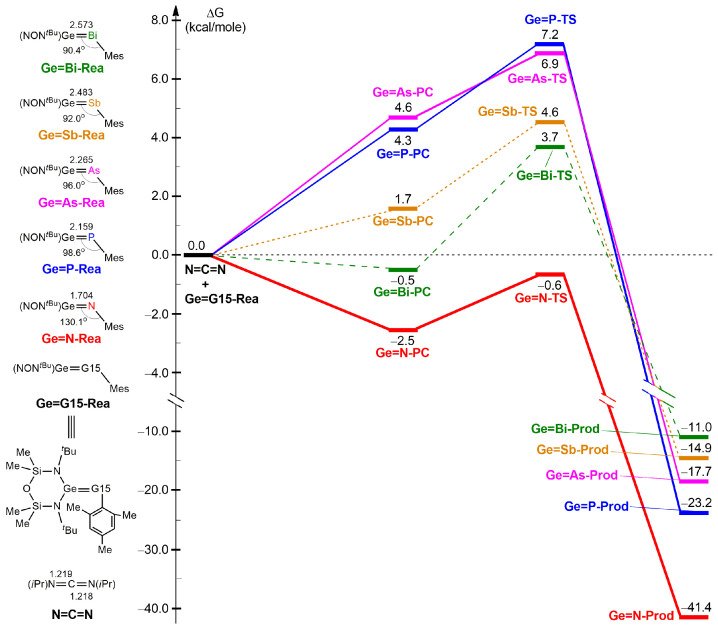
Using the M06-2X-D3/def2-TZVP method, the computed free energy profile was examined to uncover the mechanism of the [2 + 2] cycloaddition reaction between **Ge=G15-Rea** and **N=C=N**. Bond lengths are expressed in angstroms, and bond angles in degrees, with some hydrogen atoms omitted for clarity.

**Figure 3:**
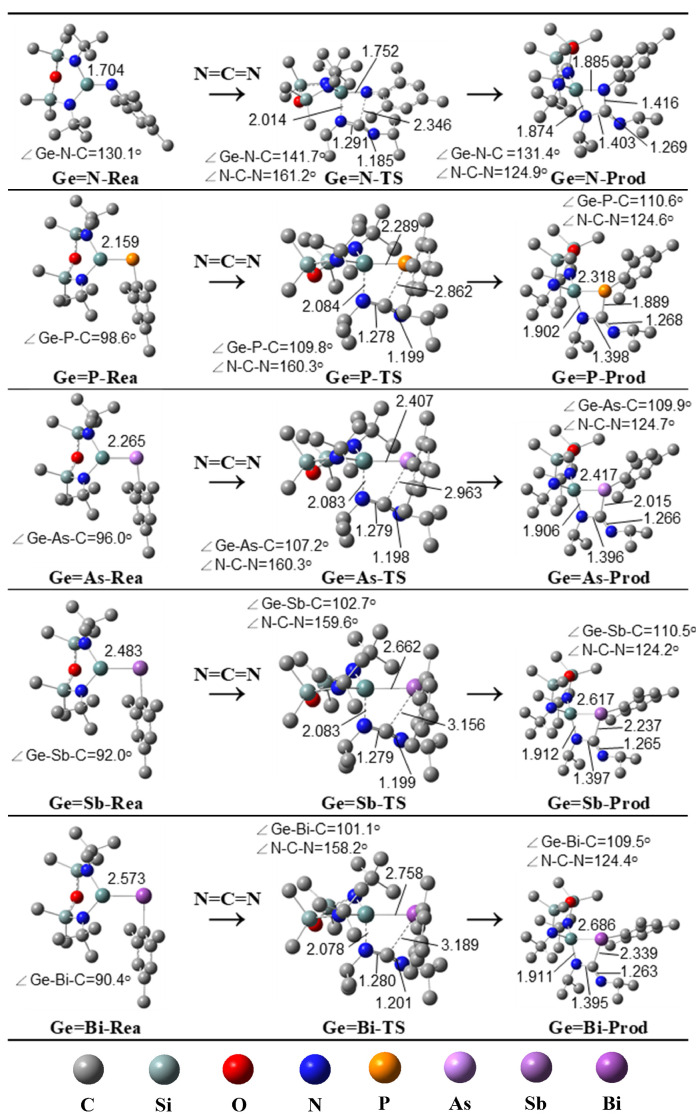
The structures of the reactants (**Ge=G15-Rea**), transition states (**Ge=G15-TS**), and products (**Ge=G15-Prod**) in the gas phase for the cycloaddition reactions with **N=C=N** were optimized using the M06-2X-D3/def2-TZVP method. Bond lengths and bond angles are expressed in angstroms (Å) and degrees (°), respectively.

Figure 2 presents the free energy profiles for the [2 + 2] cycloaddition reactions of heavy imine-like **Ge=G15-Rea** molecules with **N=C=N** (Equation (1)), computed at the M06-2X-D3/def2-TZVP level. The calculated energy profile for the reaction sequence **Ge=G15-Rea** + **N=C=N** → **Ge=G15-PC** (precursor complex) → **Ge=G15-TS** → **Ge=G15-Prod**, is also illustrated in Figure 2. The geometrically optimized structures of **Ge=G15-Rea**, **Ge=G15-TS**, and **Ge=G15-Prod** can be found in Figure 3.

As predicted by the valence bonding model illustrated in Figure 1, steric interactions among the attached ligands should cause the bond angle ∠Ge–G15–L_3_ in **Ge=G15-Rea** to exceed 90°. Our M06-2X-D3 results in Figure 3 confirm that the bond angle ∠Ge–G15–Mes in **Ge=G15-Rea** is consistently above 90° (approximately 90.4–130.1°), which agrees well with our theoretical prediction. Additionally, the present DFT results shown in Figure 3 indicate that the bond angle ∠Ge–G15–Mes decreases in the following order: 130.1° (**Ge=N-Rea**) > 98.6° (**Ge=P-Rea**) > 96.0° (**Ge=As-Rea**) > 92.0° (**Ge=Sb-Rea**) > 90.4° (**Ge=Bi-Rea**). This trend aligns well with the variation in the Ge–G15 bond length (Å) in **Ge=G15-Rea**, which follows the sequence: 1.704 (**Ge=N-Rea**) < 2.159 (**Ge=P-Rea**) < 2.265 (**Ge=As-Rea**) < 2.483 (**Ge=Sb-Rea**) < 2.573 (**Ge=Bi-Rea**). In other words, as the Ge–G15 bond length increases in **Ge=G15-Rea**, the steric repulsion between the attached ligands decreases, ultimately resulting in a smaller bond angle ∠Ge–G15–Mes in **Ge=G15-Rea**. From Figure 2, our M06-2X-D3 calculations reveal that **Ge=G15-PC** precursor complexes lie at shallow energy levels (−2.5 to 4.6 kcal/mol) relative to their corresponding reactants, making their experimental detection unlikely.

As seen in Figure 2, the computed Gibbs free activation energies (Δ*G*_ACT_) for heavy imine-like **Ge=G15-Rea** species are within −0.6–7.2 kcal/mol, suggesting relatively low barriers compared to the corresponding precursor complex, **Ge=G15-PC**. Meanwhile, their reaction free energies (Δ*G*_RXN_) remain negative (−41.4 to −11.0 kcal/mol), demonstrating a thermodynamically favorable process. More precisely, our M06-2X-D3 computations, presented in Figure 2, reveal that the Gibbs free activation barriers (Δ*G*_ACT_, kcal/mol) increase relative to **Ge=G15-PC** in the order: **Ge=N-TS** (1.9) < **Ge=P-TS** (2.4) < **Ge=As-TS** (2.7) < **Ge=Sb-TS** (2.9) < **Ge=Bi-TS** (4.2). Additionally, the Gibbs free reaction energies (Δ*G*_RXN_, kcal/mol) for the [2 + 2] cycloaddition between **N=C=N** and **Ge=G15-Rea** exhibit an increasing trend: **Ge=N-Prod** (−41.4) < **Ge=P-Prod** (−23.2) < **Ge=As-Prod** (−17.7) < **Ge=Sb-Prod** (−14.9) < **Ge=Bi-Prod** (−11.0). Our computational findings indicate that the free activation and reaction energies exhibit a trend dictated by the atomic number of the Group 15 element. Specifically, in the **Ge=G15-Rea** system, a higher atomic number of G15 corresponds to an increased activation barrier, making the [2 + 2] cycloaddition reaction with **N=C=N** less exergonic. Until now, only one experimental study has supported our M06-2X prediction that the **Ge=N-Rea** molecule [42,43], possessing a Ge=N double bond and resembling a heavy imine, can undergo this reaction with ease (Scheme 1).

### 2.3. The Interaction Models

**Figure 4:**
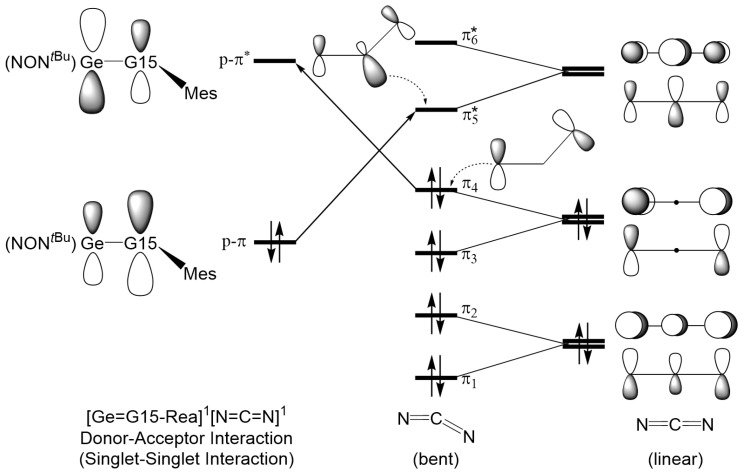
The “donor–acceptor bonding model”, also known as the “singlet–singlet interaction model”, can be represented by the reaction: [**Ge=G15-Rea**]^1^ + [**N=C=N**]^1^ → [**Ge=G15-TS**]^1^. * means anti-bonding.

**Figure 5:**
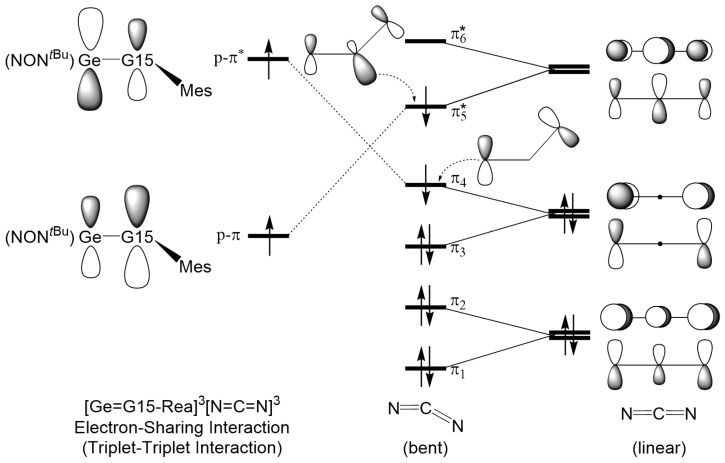
The “electron-sharing bonding model”, also known as the “triplet–triplet interaction model”, can be represented by the reaction: [**Ge=G15-Rea**]^3^ + [**N=C=N**]^3^ → [**Ge=G15-TS**]^1^. * means anti-bonding.

To better understand the bonding interactions in the [2 + 2] cycloaddition reaction between heavy imine-like **Ge=N-Rea** molecules and **N=C=N**, we introduce two bonding models, illustrated in Figure 4 and Figure 5, for analyzing the electronic interactions between **Ge=G15-Rea** and **N=C=N**. To analyze the transition state structure (**Ge=G15-TS**), we separate it into two fragments: **Ge=G15-Rea** and **N=C=N**. The interaction between their highest occupied molecular orbital (HOMO) and lowest unoccupied molecular orbital (LUMO) leads to two bonding models: “singlet–singlet (S–S)” and “triplet–triplet (T–T)” interactions. These two bonding models, shown in Figure 4 and Figure 5, both contribute to the singlet reaction.

The “singlet–singlet interaction model” shown in Figure 4 is represented by [**Ge=G15-Rea**]^1^ + [**N=C=N**]^1^ → [**Ge=G15-TS**]^1^ and is also referred to as the “donor–acceptor bonding model.” Its stability is derived from two major interactions: (the filled p-π orbital of **Ge=G15-Rea**) → (the LUMO of **N=C=N**) and (the vacant p-π* orbital of **Ge=G15-Rea**) ← (the HOMO of **N=C=N**).

The “triplet–triplet interaction model” shown in Figure 5 describes the interaction of the p-π orbital of **Ge=G15-Rea**, containing one electron, with the singly occupied molecular orbital (SOMO) of **N=C=N**. Simultaneously, the SOMO of **N=C=N** interacts with the p-π* orbital of **Ge=G15-Rea**, which also contains one electron. This bonding interaction is called the “electron-sharing model” and is represented as [**Ge=G15-Rea**]^3^ + [**N=C=N**]^3^ → [**Ge=G15-TS**]^1^.

### 2.4. The FMO and EDA–NOCV Analyses

**Figure 6:**
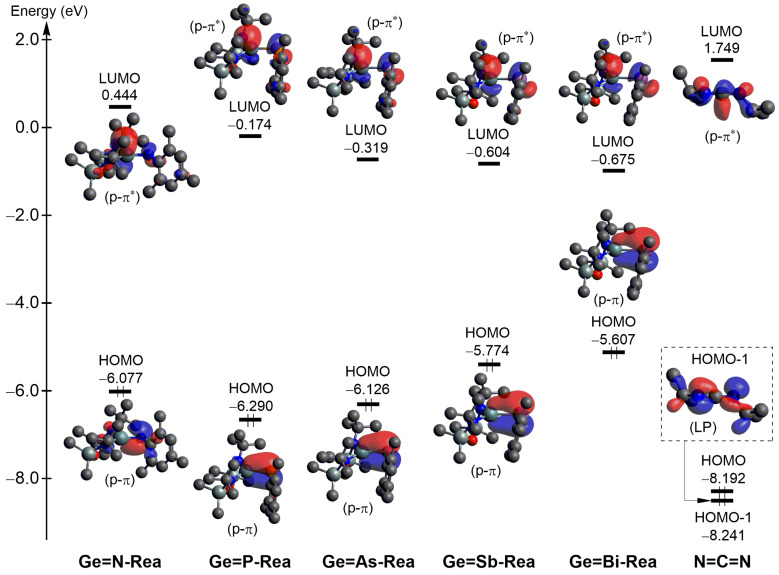
A schematic representation of the essential frontier molecular orbitals and their orbital energies (in eV) is provided for five heavy imine analogues, **Ge=G15-Rea** and **N=C=N**, with hydrogen atoms excluded for clarity. * means anti-bonding.

**Figure 7:**
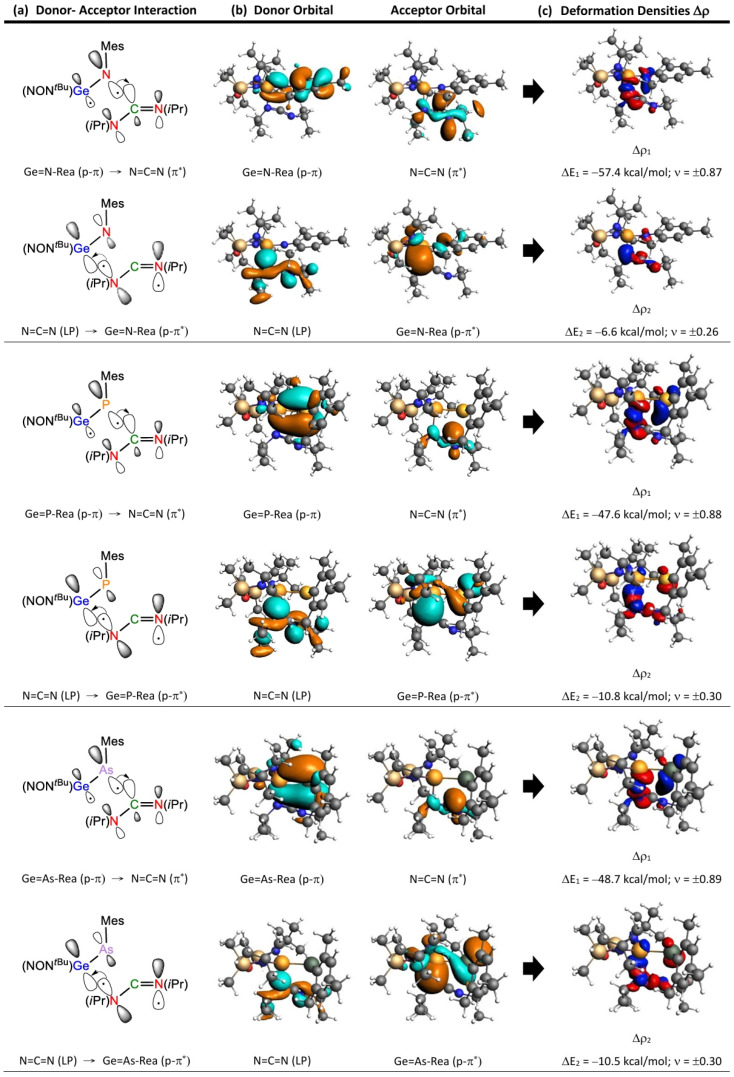

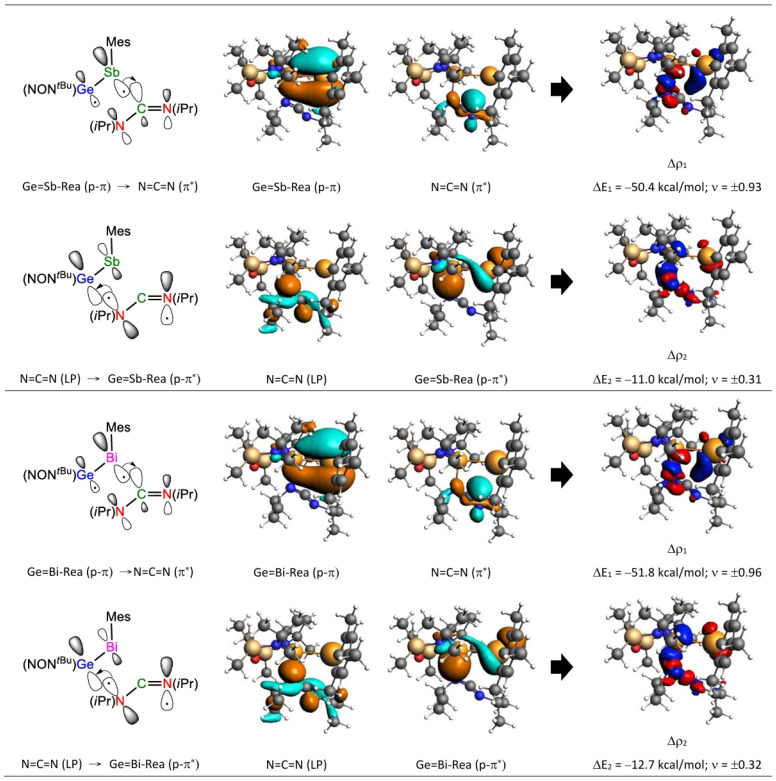
The transition states (**Ge=G15-TS**) are shown with plots of (**a**) donor–acceptor interaction, (**b**) donor orbitals and acceptors, and (**c**) electron density deformations. These plots illustrate Δρ_1_ and Δρ_2_ corresponding to ∆*E*_Orb(1)_ and ∆*E*_Orb(2)_ for both S–S and T–T models. Each subplot features red and blue contours indicating negative and positive density deformations. Hydrogen atoms are excluded for clarity, and the contour value is set at |Δρ| = 0.002 a.u. across all transition states. Computational results were obtained with the ZORA-M06-2X-D3/TZ2P//M06-2X-D3/def2-TZVP method. * means anti-bonding.

The critical frontier molecular orbitals of **N=C=N** and **Ge=G15-Rea** heavy imine analogues, including the filled p-π orbital and the empty p-π* orbital, along with their orbital energies, are collected in Figure 6. In **Ge=G15-Rea**, the p-π molecular orbital is largely localized on the more electronegative G15 atom, while the p-π* orbital is mainly centered on the less electronegative Ge element. According to Fukui’s frontier molecular orbital (FMO) theory [69], the reactivity of a chemical reaction is determined by the energy gap between the interacting molecular orbitals. As shown in Table 2, the energy gaps (in eV) between the p-π orbital of **Ge=G15-Rea** and the LUMO of **N=C=N** range from 7.356 to 8.039, which are narrower than the gaps between the HOMO-1 of **N=C=N** and the p-π* orbital of **Ge=G15-Rea**, which span 7.566 to 8.685. Consequently, our theoretical findings show that in the [2 + 2] cycloaddition reaction of **Ge=G15-Rea** with **N=C=N**, the electron transfer from the filled p-π orbital of **Ge=G15-Rea** to the LUMO of **N=C=N** (p-π (**Ge=G15-Rea**) → LUMO (**N=C=N**)) is more dominant than the electron transfer from the HOMO-1 of **N=C=N** to the vacant p-π* orbital of **Ge=G15-Rea** (p-π* (**Ge=G15-Rea**) ← HOMO-1 (**N=C=N**)). The aforementioned finding will be further substantiated using more advanced methods, which will be discussed later, with the supporting theoretical evidence presented in Table 3 and Figure 7.

To validate the FMO observations shown in Table 2, we also incorporate the EDA–NOCV results, considering the transition states, **Ge=G15-TS**, as two distinct parts: **Ge=G15-Rea** and **N=C=N**. As previously mentioned, two interaction models (S–S and T–T interaction models) were applied to explore the electronic structures of these **Ge=G15-TS** stationary points. The EDA–NOCV numerical results for the five **Ge=G15-TS** species, based on two different fragmentation schemes (Figure 4 and Figure 5), are provided in Table 3. As noted earlier [68,69], the fragmentation scheme that shows the least variation in Δ*E*_Orb_ during bond formation best represents the electronic structure of a transition state. The absolute values of Δ*E*_Orb_ (in kcal/mol) for the five **Ge=G15-TS** species in the “S–S interaction” model, as shown in Table 3, range from 80.5 to 95.4, which are significantly smaller than the absolute values in the “T–T interaction” model, where the range is from 208.8 to 291.1. Therefore, the analysis in Table 3 supports the idea that the “S–S interaction” (“donor–acceptor interaction”) model more closely represents the bonding interactions in the five **Ge=G15-TS** transition states compared to the “T–T interaction” (“electron-sharing interaction”) model. From now on, we will exclusively adopt the “S–S interaction” model rather than the “T–T interaction” model.

As displayed in Table 3, the orbital interaction energy decomposition (∆*E*_Orb_) in **Ge=G15-TS**, employing the “S–S interaction” model, reveals that ∆*E*_Orb(1)_ and ∆*E*_Orb(2)_ together constitute about 67.1% to 77.3% of the ∆*E*_Orb_. The EDA analysis given in Table 3 further dissects this energy into contributions from electron density deformation (Δρ), elucidating the nature of each interaction. The deformation density distributions of ∆ρ_1_ and ∆ρ_2_, mapped within the “S–S interaction” model and associated with ∆*E*_Orb(1)_ and ∆*E*_Orb(2)_, are shown in Figure 7. The red-to-blue color progression illustrates charge transfer directionality. The dominant interaction takes place as the filled p-π orbital of **Ge=G15-Rea** donates electron density to the vacant LUMO of **N=C=N**, with ∆*E*_Orb(1)_ representing approximately 59.1–62.1% of the ∆*E*_Orb_ term. In contrast, the ∆*E*_Orb(2)_ interaction, where the HOMO-1 of **N=C=N** transfers electrons to the unoccupied p-π* orbital of **Ge=G15-Rea**, accounts for about 6.9–15.2%. The findings provide strong support that the bonding framework of the **Ge=G15-TS** transition state is predominantly influenced by the interaction between the filled p-π orbital of **Ge=G15-Rea** and the LUMO of **N=C=N**, labeled as p-π (**Ge=G15-Rea**) → LUMO (**N=C=N**). Notably, this theoretical insight, drawn from EDA–NOCV analysis in Table 3 and Figure 7, corresponds with the FMO-based results in Table 2.

### 2.5. The ASM Analyses

**Figure 8:**
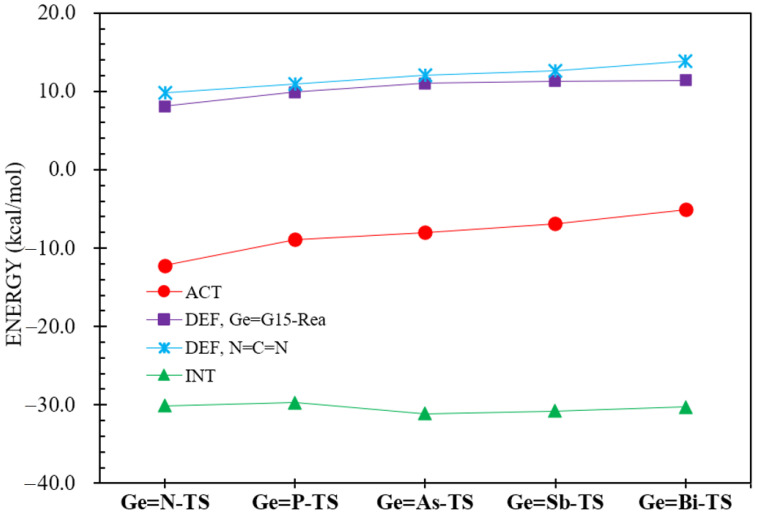
The activation energy (Δ*E*_ACT_) decomposition for the transition states (**Ge=G15-TS**) in the [2 + 2] cycloaddition reactions between **Ge=G15-Rea** and N=C=N, with data extracted from Table 4.

To determine the critical factor influencing the barrier heights of [2 + 2] cycloaddition reactions involving **Ge=G15-Rea** molecules and **N=C=N**, this study conducts an ASM analysis. Table 4 indicates that Δ*E*_ACT_ consists of two deformation energy contributions (Δ*E*_DEF,N=C=N_ + Δ*E*_DEF,Ge=G15-Rea_), along with an interaction energy (Δ*E*_INT_) between the deformed reactants. Figure 8 demonstrates that Δ*E*_INT_ remains nearly constant, irrespective of the G15 element present in **Ge=G15-TS**. Importantly, the deformation energies of both **Ge=G15-Rea** (Δ*E*_DEF,Ge=G15-Rea_) and **N=C=N** (Δ*E*_DEF,N=C=N_) fragments are the primary factors influencing the activation barrier (Δ*E*_ACT_). The reasoning behind this phenomenon is straightforward. In the [2 + 2] cycloaddition between **N=C=N** and **Ge=G15-Rea**, the N=C bond of the former interacts with the Ge=G15 bond of the latter to form a four-membered heterocyclic ring (**Ge=G15-Prod**). Consequently, both **N=C=N** and **Ge=G15-Rea** experience structural distortions, making their deformation energies key factors in determining the activation energy of the [2 + 2] cycloaddition reaction. However, achieving maximum orbital overlap demands substantial geometric adjustments. As the atomic radius of the G15 element increases, the necessary distortions become more severe, leading to higher deformation energies and activation barriers, as depicted in Figure 8.

Moreover, based on the calculated structural parameters summarized in Figure 3, the ∠Ge–G15–C bond angle in the **Ge=G15-TS** transition state expands in the order **8.9% (Ge=N-TS) < 11.4% (Ge=P-TS) < 11.6% (Ge=As-TS) < 11.7% (Ge=Sb-TS) < 11.8% (Ge=Bi-TS)**, relative to its initial value in the heavy imine analog (Ge=G15-Rea). Similarly, the ∠N–C–N bond angle in the N=C=N unit of Ge=G15-TS follows an increasing trend: **10.4% (Ge=N-TS) < 10.9% (Ge=P-TS) ≈ 10.9% (Ge=As-TS) < 11.3% (Ge=Sb-TS)**. Besides, our DFT calculations, as summarized in Figure 3, indicate a systematic decrease in the ∠N–C–N bond angle within the **N=C=N** moiety of **Ge=G15-Prod**, following the order: **Ge=N-Prod (22.5%) > Ge=P-Prod (22.3%) > Ge=As-Prod (22.2%) > Ge=Sb-Prod (22.1%) > Ge=Bi-Prod (21.4%)**, compared to its corresponding **G13=P-TS** stationary point. These computational results strongly indicate that Ge=N-TS is reactant-like, whereas Ge=Bi-TS is product-like. In other words, the theoretical findings indicate that the heavy imine-like Ge=N-Rea molecule, characterized by a Ge=N double bond, undergoes a [2 + 2] cycloaddition reaction with **N=C=N** via an earlier transition state, exhibiting the lowest activation barrier and the highest exergonicity. In contrast, the **Ge=Bi-Rea** molecule, which contains a Ge=Bi double bond, follows a reaction pathway with a later transition state, the highest activation barrier, and the lowest exergonicity. The consistency of these findings with the Hammond postulate [70] is confirmed by our M06-2X-D3 calculations, as shown in Figure 2 and Figure 3.

## 3. Methodology

The molecular geometry optimizations were carried out with the Gaussian 16 program [71] at the M06-2X [72]-D3 [71,72,73,74,75]/def2-TZVP [76] theoretical level, omitting any symmetry constraints. Subsequent frequency calculations ensured that the stationary points correspond to true energy minima on the potential energy surface.

Energy decomposition analyses (EDAs) [57,58,59,60,61,62] were carried out at the M06-2X-D3 level using the ADF 2023.104 program [77]. The analyses featured uncontracted Slater-type orbitals with two polarization functions, a triple-ζ-quality basis set, and a frozen-core approximation for the core electrons (TZ2P) [78]. Relativistic effects were incorporated into the single-point calculations using the zero-order regular approximation (ZORA) [79,80]. The EDA analyses relied on geometries optimized at the M06-2X-D3/def2-TZVP level. This computational approach is referred to as ZORA-M06-2X-D3/TZ2P//M06-2X-D3/def2-TZVP.

The EDA method was used to perform a quantitative analysis of the bonding interactions between the heavy imine-like fragment (**Ge=G15-Rea**) and dicyclohexylcarbodiimide (**N=C=N**, Scheme 2). The interaction energy (∆*E*_INT_) was further broken down into several physically relevant contributions.

Δ*E*_INT_ = Δ*E*_Elstat_ + Δ*E*_Pauli_ + Δ*E*_Orb_ + Δ*E*_Disper_ (1)

In Equation (1), Δ*E*_Elstat_ denotes the electrostatic interaction energy between the charge density distributions of **Ge=G15-Rea** and **N=C=N** (Scheme 2) in their transition state geometry, **Ge=G15-TS**. Furthermore, this term involves the doubly bonded segment of **Ge=G15-Rea**. Δ*E*_Pauli_, the second term, describes the repulsive interaction between the **Ge=G15-Rea** and **N=C=N** fragments, driven by the Pauli exclusion principle, which restricts electrons with identical spins from sharing the same spatial region. The third term in Equation (1), Δ*E*_Orb_, represents the stabilization due to orbital interactions, assessed in the last phase of the EDA following the optimization of the Kohn–Sham orbitals. This term can also be broken down into contributions from orbitals associated with different irreducible representations in the interacting system. Δ*E*_Disper_, the fourth term, accounts for the dispersion interaction between the **Ge=G15-Rea** and **N=C=N** fragments.

Furthermore, natural orbitals for chemical valence (NOCV) [63,64,65,66] analysis enables visual inspection of the deformation density (Δρ), allowing researchers to attribute the Δ*E*_Orb_ term to distinct bond types. This process illustrates the extent of charge deformation and provides a visual representation of the charge redistribution associated with pairwise orbital interactions. This allows the EDA–NOCV method to deliver both quantitative measurements and qualitative insights into the strength of orbital interactions in chemical bonds.

Additionally, to fully understand the factors affecting **N=C=N** capture reactions involving heavy imines, the research emphasized the energies required to distort the reactant fragments into the transition state geometry (Δ*E*_DEF_) and the energies arising from the interactions between these distorted reactants at that state (Δ*E*_INT_). The activation strain model (ASM) [81,82,83,84,85], an analytical framework, extends the distortion/interaction method first developed by Ess and Houk [86,87,88] and has proven effective in a variety of chemical systems.

## 4. Conclusions

By employing FMO, EDA–NOCV, and ASM approaches, this research investigates the reactivity patterns of [2 + 2] cycloaddition reactions involving the heavy imine-like molecule (**Ge=G15-Rea**) and the heterocumulene (**N=C=N**). Several noteworthy conclusions emerge from this research.

**Scheme 3:**
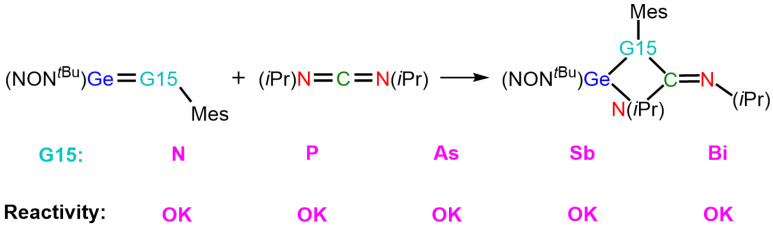
Theoretical predictions of the [2 + 2] cycloaddition reaction between **Ge=G15-Rea** and the **N=C=N** molecule.

**Scheme 4:**
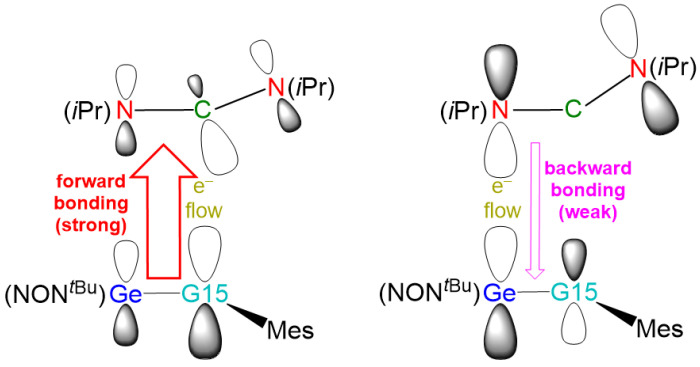
Donor–acceptor bonding interactions between the heavy imine-like molecule **Ge=G15-Rea** and the **N=C=N** molecule.

(1) Both EDA theoretical analysis and the valence-electron bonding model reveal that the Ge=G15 moiety in heavy imine-like **Ge=G15-Rea** molecules exhibits an “electron-sharing bonding interaction”.

(2) Our valence bond theory analysis suggests that steric effects between attached ligands cause the bond angle (∠Ge–G15–ligand) in heavy imine-like **Ge=G15-Rea** molecules to exceed 90°, a prediction that agrees with both experimental [42,43] and the present theoretical findings.

(3) Our M06-2X-D3 computations reveal that all heavy imine-like **Ge=G15-Rea** molecules studied here are capable of undergoing [2 + 2] cycloaddition reactions with the **N=C=N** molecule, yielding four-membered heterocyclic structures (Scheme 3). These findings are corroborated by kinetic and thermodynamic evaluations and are consistent with experimental reports on Ge=N-based imine-like molecules [42,43].

(4) Our EDA results demonstrate that the bonding interaction between **N=C=N** and the heavy imine-like molecule **Ge=G15-Rea** in **Ge=G15-TS** follows a “donor–acceptor bonding model” (“singlet–singlet bonding”) rather than an “electron-sharing bonding model” (“triplet–triplet bonding”).

(5) Both FMO and EDA–NOCV calculations indicate that the bonding in **Ge=G15-TS** is primarily characterized by electron transfer from the filled p-π orbital of **Ge=G15-Rea** to the vacant p-π* orbital of **N=C=N**, representing the dominant bonding interaction. A smaller contribution involves electron transfer from the filled p-π orbital of **N=C=N** to the empty p-π* orbital of **Ge=G15-Rea**, which is less significant (Scheme 4).

(6) The ASM analysis clearly shows that the activation barrier of the [2 + 2] cycloaddition reaction between the **N=C=N** molecule and the heavy imine-like molecule **Ge=G15-Rea** is greatly influenced by the deformation energies of both fragments.

It is hoped that our theoretical insights will inspire experimental chemists to develop more efficient and groundbreaking results.

## Figures and Tables

**Table 1:** The chemical bonding between (NONtBu)Ge: and :G15–Mes (with G15 representing a Group 15 element) in the reactant Ge=G15-Rea ((NONtBu)Ge=G15–Mes) was examined using EDA–NOCV at the ZORA-M06-2X-D3/TZ2P//M06-2X-D3/def2-TZVP level. The EDA–NOCV analysis takes into account both singlet (S) and triplet (T) spin states. All energy values are reported in kcal/mol.

Fragments	Ge=N-Rea		Ge=P-Rea		Ge=As-Rea		Ge=Sb-Rea		Ge=Bi-Rea	
	(NON*^t^*^Bu^)Ge: (S) + :N–Mes (S)	(NON*^t^*^Bu^)Ge: (T) + :N–Mes (T)	(NON*^t^*^Bu^)Ge: (S) + :P–Mes (S)	(NON*^t^*^Bu^)Ge: (T) + :P–Mes (T)	(NON*^t^*^Bu^)Ge: (S) + :As–Mes (S)	(NON*^t^*^Bu^)Ge: (T) + :As–Mes (T)	(NON*^t^*^Bu^)Ge: (S) + :Sb–Mes (S)	(NON*^t^*^Bu^)Ge: (T) + :Sb–Mes (T)	(NON*^t^*^Bu^)Ge: (S) + :Bi–Mes (S)	(NON*^t^*^Bu^)Ge: (T) + :Bi–Mes (T)
Δ*E*_INT_^(a)^	−96.0	−111.9	−83.0	−96.7	−72.9	−84.6	−58.0	−70.0	−50.3	−61.9
Δ*E*_Pauli_	490.1	346.2	445.9	195.3	422.8	176.6	323.6	145.7	295.7	130.1
Δ*E*_Elstat_^(b)^	−249.8 (42.6%)	−204.4 (44.6%)	−241.5 (45.7%)	−140.8 (48.2%)	−236.2 (47.6%)	−133.9 (51.3%)	−192.7 (50.5%)	−117.4 (54.4%)	−180.4 (52.1%)	−108.9 (56.7%)
Δ*E*_Orb_^(b)^	−334.4 (57.1%)	−251.8 (55.0%)	−285.5 (54.0%)	−149.3 (51.1%)	−257.7 (52.0%)	−125.4 (48.0%)	−187.0 (49.0%)	−96.5 (44.7%)	−163.8 (47.4%)	−81.3 (42.3%)

^(a)^ Δ*E*_INT_ = Δ*E*_Elstat_ + Δ*E*_Pauli_ + Δ*E*_Orb_. ^(b)^ The values in parentheses offer the percentage contribution to the total attractive interactions (Δ*E*_Elstat_ + Δ*E*_Orb_).

**Table 2:** Energy differences (in eV) between the critical molecular orbitals of **Ge=G15-Rea** and **N=C=N** in the [2 + 2] cycloaddition reaction, computed at the M06-2X-D3/def2-TZVP level, are shown. The data are taken from Figure 6.

System	Energy Difference N=C=N (LUMO)—Ge=G15-Rea (HOMO)	Energy Difference Ge=G15-Rea (LUMO)—N=C=N (HOMO-1)
**Ge=N-Rea** + **N=C=N**	7.826	8.685
**Ge=P-Rea** + **N=C=N**	8.039	8.067
**Ge=As-Rea** + **N=C=N**	7.875	7.922
**Ge=Sb-Rea** + **N=C=N**	7.523	7.637
**Ge=Bi-Rea** + **N=C=N**	7.356	7.566

**Table 3:** The transition state (**Ge=G15-TS**) bonding between **Ge=G15-Rea** (G15 = Group 15 element) and **N=C=N** is explored using EDA–NOCV results at the ZORA-M06-2X-D3/TZ2P//M06-2X-D3/def2-TZVP level, with both singlet and triplet spin states considered. Energy values are given in kcal/mol.

Fragments	Ge=N-TS		Ge=P-TS		Ge=As-TS		Ge=Sb-TS		Ge=Bi-TS	
	Ge=N-Rea (S) + N=C=N (S)	Ge=N-Rea (T) + N=C=N (T)	Ge=P-Rea (S) + N=C=N (S)	Ge=P-Rea (T) + N=C=N (T)	Ge=As-Rea (S) + N=C=N (S)	Ge=As-Rea (T) + N=C=N (T)	Ge=Sb-Rea (S) + N=C=N (S)	Ge=Sb-Rea (T) + N=C=N (T)	Ge=Bi-Rea (S) + N=C=N (S)	Ge=Bi-Rea (T) + N=C=N (T)
Δ*E*_INT_^(a)^	−30.1	−172.8	−29.7	−155.0	−31.1	−148.1	−30.8	−139.4	−30.3	−131.1
Δ*E*_Pauli_	207.0	291.4	175.0	236.6	173.7	232.6	175.2	223.1	178.5	219.0
Δ*E*_Elstat_^(b)^	−138.7 (58.5%)	−170.2 (36.7%)	−121.4 (59.3%)	−144.0 (36.8%)	−121.1 (59.1%)	−142.6 (37.4%)	−121.2 (58.9%)	−139.1 (38.4%)	−122.8 (58.8%)	−138.6 (39.6%)
Δ*E*_Orb_^(b)^	−95.4 (40.2%)	−291.1 (62.7%)	−80.5 (39.3%)	−244.8 (62.5%)	−81.0 (39.5%)	−235.4 (61.8%)	−82.1 (39.8%)	−220.6 (60.9%)	−83.4 (39.9%)	−208.8 (59.6%)
Δ*E*_Orb(1)_^(c)^	−57.4 (60.2%)	−234.1 (80.4%)	−47.6 (59.1%)	−199.6 (81.6%)	−48.7 (60.1%)	−195.7 (83.1%)	−50.4 (61.4%)	−182.9 (82.9%)	−51.8 (62.1%)	−170.2 (81.5%)
Δ*E*_Orb(2)_^(c)^	−6.6 (6.9%)	−27.2 (9.4%)	−10.8 (13.4%)	−24.6 (10.1%)	−10.5 (12.9%)	−23.8 (10.1%)	−11.0 (13.4%)	−24.5 (11.1%)	−12.7 (15.2%)	−26.8 (12.8%)
Δ*E*_Rest_^(c)^	−31.4 (32.9%)	−29.7 (10.2%)	−22.1 (27.5%)	−20.5 (8.4%)	−21.8 (27.0%)	−15.9 (6.7%)	−20.7 (25.2%)	−13.3 (6.0%)	−18.9 (22.7%)	−11.8 (5.7%)
Δ*E*_Disper_^(b)^	−3.0 (1.3%)	−3.0 (0.6%)	−2.8 (1.4%)	−2.8 (0.7%)	−2.8 (1.3%)	−2.8 (0.7%)	−2.7 (1.3%)	−2.7 (0.7%)	−2.7 (1.3%)	−2.7 (0.8%)

^(a)^ Δ*E*_INT_ = Δ*E*_Elstat_ + Δ*E*_Pauli_ + Δ*E*_Orb_ + Δ*E*_Disper_. ^(b)^ The percentages shown in parentheses indicate the contribution to the total attractive interactions, Δ*E*_Elstat_ + Δ*E*_Orb_ + Δ*E*_Disper_. ^(c)^ The percentages shown in parentheses indicate the contribution to the total orbital interactions, Δ*E*_Orb_.

**Table 4:** The [2 + 2] cycloaddition reactions between **Ge=G15-Rea** and **N=C=N** were analyzed using energy decomposition analysis, with optimization at the ZORA-M06-2X-D3/TZ2P//M06-2X-D3/def2-TZVP level of theory.

Entry	Ge=N-TS	Ge=P-TS	Ge=As-TS	Ge=Sb-TS	Ge=Bi-TS
Δ*E*_ACT_^(a,b)^	−12.2	−8.9	−8.0	−6.9	−5.1
Δ*E*_DEF,N=C=N_	9.8	10.9	12.1	12.6	13.8
Δ*E*_DEF,Ge=G15-Rea_	8.1	9.9	11.0	11.3	11.4
Δ*E*_INT_	−30.1	−29.7	−31.1	−30.8	−30.3

^(a)^ Δ*E*_ACT_ = Δ*E*_DEF,N=C=N_ + Δ*E*_DEF,Ge=G15-Rea_ + Δ*E*_INT_. ^(b)^ All in kcal mol^−1^.

## Data Availability

Data is contained within the article and Supplementary Materials.

## Supplementary Materials

The following supporting information can be downloaded at: https://www.mdpi.com/article/10.3390/molecules30091905/s1. The optimized geometries calculated for all stationary points (Ge=G15-Rea, Ge=G15-TS, Ge=G15-Prod) using the M06-2X-D3/def2-TZVP level of theory are collected in the Supporting Information.
