# Supplementary material for: Insights into the Factors Controlling the Origin of Activation Barriers in the [2 + 2] Cycloaddition Reactions of Heavy Imine-like Molecules Featuring a Ge=Group 15 Double Bond with Heterocumulenes
Source: Molecules. 2025 Apr 25;30(9):1905. doi: 10.3390/molecules30091905 (PMC12073406; doi:10.3390/molecules30091905)
Supplement: Supplementary file 1 [file molecules-30-01905-s001.zip › molecules-3575057-supplementary.pdf]

Supporting Information

Insights Into the Factors Controlling the Origin of Activation Barriers in the [2 + 2] Cycloaddition Reactions of Heavy Imine-like Molecules Featuring a Ge=Group 15 Double Bond with Heterocumulenes

Zheng-Feng Zhang[1] and Ming-Der Su[1,2]*

[1]Department of Applied Chemistry, National Chiayi University, Chiayi 60004, Taiwan
[2]Department of Medicinal and Applied Chemistry, Kaohsiung Medical University, Kaohsiung 80708, Taiwan

*E-mail: midesu@mail.ncyu.edu.tw

Contents

1. The computed singlet-triplet energy gaps for $(L_1L_2)Ge:$ and $:G15–L_3$ fragments orbitals (Table S1).. S3
2. Optimized Cartesian coordinates for the **Ge=G15-Rea**, **Ge=G15-PC**, **Ge=G15-TS**, **Ge=G15-Prod** species (Table S2–S22) …... S4

Table S1. The computed singlet-triplet energy splittings for $(L_1L_2)Ge$: and :G15–L_3 fragments orbitals, based on the M06-2X/def2-TZVP level of theory. G14 = Group 14 element. ΔE_1 = [E(triplet state for $(L_1L_2)Ge$:) – E(singlet state for $(L_1L_2)Ge$:)] and ΔE_2 = [E(triplet state for :G15–L_3) – E(singlet state for :G15–L_3)]. L_1L_2 = $O(SiMe_2N\textit{t}Bu)_2$ = NON^{tBu}, and L_3 = 2,4,6-$Me_3C_6H_2$ = 2,4,6-trimethylphenyl = Mes.

============================

$(NON^{tBu})Ge$:, ΔE_1 = 42.7 kcal/mol

:N–Mes, ΔE_2 = −28.6 kcal/mol

:P–Mes, ΔE_2 = −30.9 kcal/mol

:As–Mes, ΔE_2 = −31.0 kcal/mol

:Sb–Mes, ΔE_2 = −29.1 kcal/mol

:Bi–Mes, ΔE_2 = −29.4 kcal/mol

============================

(All were calculated at the M06-2X-D3/def2-TZVP level)

Table S2

Ge=N-Rea

Atomic Number	Coordinates (Angstroms) X	Y	Z
Si	-0.04384300	0.00318000	-0.01218700
O	-0.70985900	-1.21446900	0.91736300
Si	-1.45442900	-2.67686000	0.59225100
N	-0.20633100	-3.48138500	-0.35481900
Ge	1.41335400	-2.63386500	-0.37143900
N	1.28480700	-0.85374200	-0.79335000
C	2.34417300	-0.17652600	-1.59571700
C	2.93864200	-1.15176500	-2.61694200
H	3.50373900	-1.95368000	-2.11905100
H	3.64035300	-0.62509500	-3.27908700
H	2.14281500	-1.59367300	-3.23778400
C	3.46466000	0.35238400	-0.69417100
H	3.06260600	1.03361000	0.07002800
H	4.20932400	0.90281500	-1.28865900
H	3.98380300	-0.47605600	-0.19117800
C	1.71529200	0.99181700	-2.36400300
H	0.89374600	0.64194800	-3.00578000
H	2.47421700	1.47159600	-2.99762900
H	1.32711200	1.76471500	-1.68230400
N	2.78714100	-3.59621700	-0.06588900
C	4.11417900	-3.23462200	0.08506400
C	5.06944600	-3.71477700	-0.84610500
C	6.41591100	-3.39308200	-0.68412400
H	7.13709300	-3.76970700	-1.41606800
C	6.87212400	-2.61985700	0.38808400
C	5.92689500	-2.18384400	1.31857500
H	6.25894400	-1.59680200	2.18009400
C	4.56669800	-2.47398500	1.19136900
C	3.57541000	-1.98915700	2.21290300
H	4.08322000	-1.52089300	3.06624300

H	2.95303300	-2.81926800	2.57996900
H	2.88680300	-1.23830900	1.78411800
C	8.33170800	-2.26962500	0.52468600
H	8.97159800	-3.14533200	0.34300200
H	8.55616900	-1.88782900	1.52987300
H	8.62719500	-1.49407300	-0.19954800
C	4.60113100	-4.59299500	-1.97246500
H	5.44235000	-4.93117300	-2.59167600
H	3.87796600	-4.07224400	-2.62131500
H	4.07205000	-5.47206800	-1.57260600
C	-0.26668200	-4.92092100	-0.74755400
C	0.51080700	-5.13088700	-2.05240000
H	0.12348000	-4.46116300	-2.83593800
H	0.40152200	-6.16888000	-2.39819500
H	1.58356100	-4.93944700	-1.90286800
C	0.31803900	-5.81059300	0.35619700
H	1.36769400	-5.53988600	0.53811600
H	0.26763700	-6.87051500	0.06403000
H	-0.24855600	-5.68466100	1.29104500
C	-1.72758100	-5.30815500	-0.99965500
H	-2.33474500	-5.22858700	-0.08423100
H	-1.78015000	-6.35445400	-1.33152700
H	-2.17476900	-4.67292400	-1.77821700
C	-1.79040800	-3.47701700	2.24549300
H	-0.84909300	-3.60058200	2.80074300
H	-2.27193400	-4.46121200	2.15642800
H	-2.45356800	-2.82643800	2.83570800
C	-3.05224800	-2.35601000	-0.33087200
H	-3.59119700	-1.53446300	0.16614700
H	-3.71101600	-3.23542500	-0.33592000
H	-2.86325400	-2.06280700	-1.37343300
C	0.50421600	1.34996800	1.15785000
H	-0.37334200	1.72688300	1.70489300
H	0.97329900	2.20077900	0.64353000
H	1.21826000	0.94979500	1.89226700
C	-1.35287900	0.63120700	-1.19760200
H	-1.52547300	-0.09170700	-2.00847100
H	-1.08029700	1.59408300	-1.65136200

H	-2.29927300	0.76963700	-0.65228500

Table S3

Ge=P-Rea

Atomic Number	Coordinates (Angstroms) X	Y	Z
Si	0.42295200	-0.26481600	0.03040000
O	-0.21760300	-1.40159200	1.08579400
Si	-1.26911200	-2.68991700	0.83406800
N	-0.41712100	-3.50869000	-0.46507900
Ge	1.38902800	-3.05045500	-0.42805700
N	1.54371800	-1.26762600	-0.88258800
C	2.50728700	-0.74160900	-1.88438500
C	2.93070000	-1.85919700	-2.83971500
H	3.48301700	-2.64089600	-2.29805700
H	3.59517300	-1.46348500	-3.62103200
H	2.05121400	-2.30980700	-3.32793400
C	3.75650300	-0.16406900	-1.20979400
H	3.48447700	0.61823600	-0.48640100
H	4.42664000	0.28203400	-1.96081500
H	4.30829700	-0.94917000	-0.67827700
C	1.82757400	0.36230400	-2.70457500
H	0.90038600	-0.00657800	-3.16688700
H	2.50291400	0.71273800	-3.49811700
H	1.58500200	1.23442300	-2.07675300
P	2.83073400	-4.47842900	0.30988100
C	4.30385100	-3.35475400	0.42189300
C	5.38326900	-3.53631200	-0.47191800
C	6.52110000	-2.73271100	-0.34999700
H	7.34481100	-2.87231000	-1.05545800
C	6.64060000	-1.77157000	0.65482600
C	5.58856100	-1.64081000	1.56320600
H	5.67372100	-0.91185100	2.37395200
C	4.42470800	-2.40950500	1.46800000
C	3.33586500	-2.20095200	2.49018300

H	3.67546000	-1.51856300	3.28090400
H	3.03250100	-3.15435500	2.94601500
H	2.42392200	-1.77579700	2.04095100
C	7.85740400	-0.88911300	0.74755000
H	8.73720200	-1.37377900	0.30348800
H	8.08940700	-0.63903800	1.79179700
H	7.69324300	0.05810900	0.21007800
C	5.35028900	-4.60441700	-1.53656300
H	6.25024500	-4.55944000	-2.16397000
H	4.46308600	-4.51715900	-2.17981000
H	5.29469400	-5.60391000	-1.07844100
C	-0.85125500	-4.75770300	-1.13410200
C	-0.27627100	-4.78823200	-2.55528900
H	-0.59038800	-3.89118300	-3.10978400
H	-0.62084200	-5.67988500	-3.09900100
H	0.82561500	-4.82327500	-2.53238000
C	-0.39071400	-6.00203400	-0.36239500
H	0.70676600	-6.06471700	-0.32618500
H	-0.76685600	-6.91858300	-0.84194500
H	-0.76480700	-5.97286100	0.67185800
C	-2.37991000	-4.77688600	-1.22933300
H	-2.84008200	-4.83012000	-0.22976000
H	-2.71137000	-5.66319800	-1.78871800
H	-2.75334300	-3.87858600	-1.74152200
C	-1.32917500	-3.66481900	2.42548100
H	-0.32185700	-4.03373500	2.67104300
H	-2.00475300	-4.53010900	2.34800400
H	-1.68087500	-3.03146100	3.25360200
C	-2.97147700	-2.02418300	0.41859300
H	-3.19822200	-1.20603600	1.12046600
H	-3.75134400	-2.78962400	0.53498400
H	-3.02780600	-1.62480300	-0.60306300
C	1.24178800	1.06993700	1.04694600
H	0.50302800	1.49758000	1.74167100
H	1.63027100	1.88639600	0.42052500
H	2.07395000	0.65776100	1.63534700
C	-0.98536500	0.45058300	-0.98258900
H	-1.34872100	-0.29319000	-1.70758300

H	-0.68980400	1.35231100	-1.53628700
H	-1.81860100	0.72020000	-0.31530700

Table S4

Ge=As-Rea

Atomic Number	Coordinates (Angstroms) X	Y	Z
Si	0.45585000	-0.29859000	0.02351400
O	-0.18981200	-1.42139300	1.09117100
Si	-1.27140100	-2.68714200	0.85215000
N	-0.45982700	-3.51029100	-0.47011100
Ge	1.36120000	-3.10182200	-0.44651500
N	1.55009900	-1.32154300	-0.90044300
C	2.51366200	-0.80985000	-1.91006700
C	2.93929800	-1.93988300	-2.84959400
H	3.49190100	-2.71422800	-2.29777700
H	3.60291200	-1.55260600	-3.63583200
H	2.06109500	-2.39841300	-3.33297700
C	3.76267300	-0.22172700	-1.24461100
H	3.49280800	0.57962100	-0.54186000
H	4.43764500	0.20254500	-2.00391900
H	4.30784900	-0.99662600	-0.69218400
C	1.83233800	0.28172600	-2.74549700
H	0.90258000	-0.09324500	-3.19779500
H	2.50495500	0.61900300	-3.54709100
H	1.59315100	1.16367000	-2.13030900
As	2.84985200	-4.61686600	0.34104800
C	4.37965100	-3.36738900	0.45740600
C	5.46395400	-3.51421100	-0.43389900
C	6.56956400	-2.66494000	-0.31556700
H	7.40037900	-2.77766200	-1.01748600
C	6.64684200	-1.68983700	0.67943900
C	5.58615200	-1.59001100	1.58145200
H	5.63838400	-0.84798700	2.38312900
C	4.45389100	-2.40637200	1.49128000

C	3.35487200	-2.22274000	2.50825000
H	3.66384400	-1.50838500	3.28325700
H	3.09280200	-3.17672500	2.98771400
H	2.42585100	-1.84862500	2.04919400
C	7.82699800	-0.75841800	0.76732600
H	8.72426300	-1.20718600	0.32059400
H	8.05209800	-0.49813000	1.81060500
H	7.62199800	0.18077200	0.22982300
C	5.48001600	-4.58601500	-1.49593000
H	6.37825000	-4.50221900	-2.12184800
H	4.59150600	-4.53812600	-2.14143300
H	5.47009500	-5.58672400	-1.03741200
C	-0.93589700	-4.73269400	-1.15793600
C	-0.40054100	-4.73540600	-2.59485700
H	-0.71545700	-3.81975900	-3.11742400
H	-0.77190600	-5.60808100	-3.15165900
H	0.70120900	-4.78564600	-2.60298200
C	-0.47748000	-6.00534900	-0.43241400
H	0.61828700	-6.10084700	-0.44935600
H	-0.89913500	-6.90126000	-0.91299900
H	-0.80333800	-5.98764000	0.61828300
C	-2.46666200	-4.72348800	-1.21043500
H	-2.89968000	-4.79455100	-0.19986800
H	-2.82899000	-5.58921700	-1.78274100
H	-2.83838500	-3.80630700	-1.68914100
C	-1.31878500	-3.67338600	2.43673400
H	-0.31221700	-4.05596700	2.66411800
H	-2.00571900	-4.53001200	2.36212600
H	-1.65071400	-3.04315400	3.27533300
C	-2.96873500	-1.98558200	0.47651700
H	-3.16313600	-1.16775300	1.18844400
H	-3.76099900	-2.73610400	0.60565200
H	-3.03936400	-1.57823600	-0.54104200
C	1.30689200	1.02638000	1.02617200
H	0.58586900	1.45543900	1.73845600
H	1.68708200	1.84297700	0.39494600
H	2.14857200	0.60528400	1.59450400
C	-0.95392400	0.43386800	-0.97541200

H	-1.33862700	-0.30859300	-1.69072500
H	-0.65128800	1.32720600	-1.53876100
H	-1.77387300	0.72117400	-0.29909000

Table S5

Ge=Sb-Rea

Atomic Number	Coordinates (Angstroms) X	Y	Z
Si	0.52823100	-0.35049200	0.00358800
O	-0.13884600	-1.44458600	1.08717100
Si	-1.27622800	-2.66212600	0.86674600
N	-0.54191500	-3.50112500	-0.49076800
Ge	1.30467000	-3.19233700	-0.51518500
N	1.56032400	-1.41277600	-0.94806100
C	2.53187300	-0.92708900	-1.96394000
C	2.95826300	-2.07385000	-2.88294400
H	3.51290000	-2.83894300	-2.32033300
H	3.61833200	-1.69650400	-3.67694000
H	2.08146700	-2.54399900	-3.35807700
C	3.78310300	-0.33586500	-1.30560800
H	3.52304900	0.50052700	-0.64169300
H	4.47687800	0.04149100	-2.07275200
H	4.30293300	-1.09864200	-0.71372200
C	1.85931700	0.15354000	-2.82071100
H	0.92548800	-0.22260700	-3.26389000
H	2.53360500	0.47059100	-3.62929300
H	1.62736700	1.04824600	-2.22165900
Sb	2.89714600	-4.89203000	0.34760600
C	4.51244500	-3.41419300	0.50380100
C	5.61676500	-3.47913200	-0.37301100
C	6.65318600	-2.54700800	-0.24764800
H	7.50040900	-2.60062200	-0.93704900
C	6.63918200	-1.55778800	0.73640100
C	5.55921700	-1.52974800	1.61899600
H	5.53990700	-0.77482500	2.41010900

C	4.49524400	-2.43501700	1.52262700
C	3.37102300	-2.30826000	2.52310800
H	3.62576000	-1.56879800	3.29444700
H	3.15803700	-3.26876100	3.01370200
H	2.42851900	-1.99277500	2.04734400
C	7.74242400	-0.53666600	0.82641400
H	8.69838200	-0.94953800	0.47690700
H	7.87386400	-0.18129400	1.85732700
H	7.51176600	0.33994900	0.20073900
C	5.73232100	-4.54207400	-1.43963500
H	6.64628300	-4.40366600	-2.03222800
H	4.86915000	-4.53379100	-2.12115700
H	5.76367600	-5.54741400	-0.99250900
C	-1.09558300	-4.68871600	-1.18003900
C	-0.64328000	-4.66917500	-2.64534900
H	-0.96041600	-3.73167400	-3.12567800
H	-1.06959400	-5.51686400	-3.20161000
H	0.45456900	-4.74688500	-2.71870900
C	-0.63737800	-5.99446400	-0.51478900
H	0.44953400	-6.13402600	-0.61820500
H	-1.12470900	-6.86368200	-0.98186700
H	-0.88461200	-5.98896200	0.55725500
C	-2.62598400	-4.63572000	-1.14513700
H	-3.00410200	-4.71582800	-0.11340800
H	-3.04449300	-5.47840100	-1.71352300
H	-2.99692800	-3.69900900	-1.58465000
C	-1.31760500	-3.66527600	2.44119900
H	-0.31856800	-4.08136300	2.64183100
H	-2.03313700	-4.49887800	2.37455300
H	-1.60983300	-3.03407000	3.29378700
C	-2.95426600	-1.88646700	0.55380200
H	-3.08805900	-1.06727600	1.27806900
H	-3.77356600	-2.60312600	0.70478500
H	-3.04158200	-1.46689600	-0.45749600
C	1.45090200	0.93830800	0.99039300
H	0.76836800	1.36594300	1.74049400
H	1.81982200	1.75988100	0.35911600
H	2.30727500	0.48929700	1.51401600

C	-0.87503000	0.43660800	-0.96325800
H	-1.30644700	-0.29251800	-1.66564300
H	-0.55145200	1.31515200	-1.53808300
H	-1.66606600	0.75879300	-0.26843900

Table S6

Ge=Bi-Rea

Atomic Number	Coordinates (Angstroms) X	Y	Z
Si	0.54380400	-0.36390700	-0.01268600
O	-0.12724500	-1.44643300	1.07912400
Si	-1.27591700	-2.65218000	0.86608000
N	-0.57259900	-3.49077600	-0.50767300
Ge	1.28393700	-3.21975600	-0.56940900
N	1.55720800	-1.43485700	-0.97470300
C	2.54195600	-0.95194400	-1.97999800
C	2.97759100	-2.09886900	-2.89494800
H	3.52941900	-2.86320500	-2.32852700
H	3.64280300	-1.71934300	-3.68357900
H	2.10611000	-2.57139600	-3.37769200
C	3.78870000	-0.36522400	-1.30919200
H	3.52871400	0.48210500	-0.65967100
H	4.49787800	-0.00458700	-2.07027600
H	4.29111800	-1.12674300	-0.70080600
C	1.88089100	0.13014400	-2.84389500
H	0.95113500	-0.24443200	-3.29702700
H	2.56434100	0.44655100	-3.64509500
H	1.64371900	1.02478200	-2.24684000
Bi	2.90635500	-5.00629700	0.32538600
C	4.56315600	-3.43323500	0.51334200
C	5.67113300	-3.46531400	-0.35767100
C	6.68210400	-2.50566000	-0.22459700
H	7.53436300	-2.53373300	-0.90954600
C	6.63493200	-1.51946400	0.76149800
C	5.54820700	-1.52176800	1.63638600

H	5.50223900	-0.76757300	2.42735700
C	4.50979500	-2.45624100	1.53052400
C	3.36968800	-2.35562600	2.51661200
H	3.59815900	-1.61428000	3.29453000
H	3.16697100	-3.32124100	3.00100700
H	2.42901000	-2.05500700	2.02708100
C	7.70910400	-0.46826000	0.86020800
H	8.67816000	-0.85364500	0.51524500
H	7.82509600	-0.11212100	1.89276800
H	7.45820600	0.40352200	0.23551100
C	5.81834600	-4.51943200	-1.42973900
H	6.72462700	-4.34890800	-2.02600800
H	4.95218100	-4.53573100	-2.10742800
H	5.88601800	-5.52540400	-0.98797700
C	-1.14851300	-4.67579400	-1.18251900
C	-0.74320600	-4.65240500	-2.66170500
H	-1.07640100	-3.71390700	-3.12867100
H	-1.18600800	-5.49913700	-3.20659400
H	0.35185700	-4.72732200	-2.77067500
C	-0.66990200	-5.98490000	-0.53693300
H	0.41204800	-6.12706600	-0.68430100
H	-1.17546800	-6.85208500	-0.98801400
H	-0.87717800	-5.98131000	0.54352500
C	-2.67733500	-4.62519300	-1.10063700
H	-3.02428300	-4.70598400	-0.05809700
H	-3.11181000	-5.46789500	-1.65694000
H	-3.06236800	-3.68883000	-1.52848200
C	-1.30311100	-3.66485400	2.43558200
H	-0.30384000	-4.08800300	2.62008600
H	-2.02531800	-4.49322700	2.37630500
H	-1.57829100	-3.03544400	3.29518500
C	-2.95208600	-1.86038700	0.58351100
H	-3.06842100	-1.04493200	1.31495000
H	-3.77569200	-2.57073900	0.74075200
H	-3.04939000	-1.43275500	-0.42359100
C	1.48437600	0.91508200	0.97017200
H	0.81030300	1.33939800	1.72980100
H	1.85063000	1.73981000	0.34164200

H	2.34334100	0.45808000	1.48271400
C	-0.85862600	0.43542900	-0.97105800
H	-1.30707200	-0.29461900	-1.66183800
H	-0.52832500	1.30320700	-1.55832600
H	-1.63802700	0.77598200	-0.27187300

Table S7

N=C=N

Atomic Number	Coordinates (Angstroms) X	Y	Z
N	-2.56345900	-1.96896300	1.55310800
C	-2.35361200	-3.39427000	1.77900600
C	-3.39294600	-1.43299300	0.83990100
C	-2.67892300	-3.71585700	3.23444300
C	-3.14419300	-4.26679500	0.81064800
H	-1.27757100	-3.57361400	1.62358100
N	-4.23998500	-0.81065700	0.22203400
H	-3.75106900	-3.55902300	3.42481300
H	-2.43163000	-4.76228900	3.46258200
H	-2.11046400	-3.06124400	3.90765900
H	-4.22490600	-4.10223500	0.94339700
H	-2.93384300	-5.33111900	0.98615500
H	-2.88497200	-4.03124000	-0.23205000
C	-4.16381800	-0.34114300	-1.15870600
C	-5.12104500	-1.16288200	-2.01545900
C	-4.50952300	1.14214800	-1.19338800
H	-3.13721100	-0.47713500	-1.54332600
H	-4.85186200	-2.22809900	-1.98918300
H	-6.14677900	-1.05790100	-1.63343900
H	-5.09594700	-0.82034300	-3.05978800
H	-4.46812700	1.52405300	-2.22335900
H	-5.52403800	1.29652500	-0.79843000
H	-3.80897200	1.71925800	-0.57518800

Table S8

Ge=N-PC

Atomic Number	Coordinates (Angstroms) X	Y	Z
Ge	0.27919600	-0.42484700	0.19625000
C	-0.26791500	1.74765500	-1.46579700
C	-2.55378000	0.23697800	0.11555100
N	-1.41611800	-0.46185500	-0.16036900
N	0.80179300	1.28929400	-0.95591800
C	2.07261500	1.65415200	-1.65819500
N	1.33954600	-1.65290600	-0.71673600
N	0.99017400	-0.22595600	1.91555300
C	2.14342600	0.95366400	-3.00915200
C	2.19815800	3.16705300	-1.79063200
H	2.86959700	1.27632500	-1.00827900
N	-1.24960500	2.25409600	-1.87016900
C	0.69312800	-2.73209300	-1.52032000
Si	3.02375000	-1.69308400	-0.22154900
C	0.24608200	-0.57723600	3.16355300
Si	2.62859400	0.39683200	1.94943100
C	-3.78257300	-0.36535700	-0.32129200
C	-2.66594100	1.51889000	0.73732800
H	1.34143800	1.31331200	-3.67507000
H	3.10810200	1.15594100	-3.49655600
H	2.02730200	-0.12957800	-2.87517600
H	1.39703300	3.57625100	-2.42639300
H	3.16015800	3.42054100	-2.25739000
H	2.14945200	3.65918700	-0.81008800
C	-2.49649100	2.17488500	-2.60631400
C	-0.17149700	-3.60818700	-0.60326300
C	-0.17274900	-2.13259800	-2.63482500
C	1.75223300	-3.61505700	-2.18983300
C	3.40174500	-3.02917000	1.04255700
C	4.31362800	-1.74428400	-1.58003300
O	3.24682100	-0.19701600	0.50973000
C	-1.24291000	-0.85254800	2.93109800

C	0.84476500	-1.85724900	3.76645900
C	0.35634600	0.57851900	4.16896600
C	2.66575500	2.26533500	1.85368200
C	3.78229800	-0.17520700	3.31388100
C	-3.73319500	-1.76273700	-0.87263500
C	-4.99014800	0.31347200	-0.23348500
C	-1.48729000	2.27579100	1.30510500
C	-3.91414800	2.15326600	0.81854900
C	-2.63747500	3.40625200	-3.49157700
C	-2.57220800	0.86056500	-3.36960600
H	-3.27712300	2.18733500	-1.82741700
H	0.44558300	-4.03157500	0.20500000
H	-0.62977500	-4.43731100	-1.16377100
H	-0.97731200	-3.00265100	-0.16291800
H	0.44718900	-1.55664300	-3.33820900
H	-0.67499200	-2.93402600	-3.19814700
H	-0.93577600	-1.47540800	-2.20004400
H	2.41265600	-4.10285900	-1.45636900
H	1.25077800	-4.41144600	-2.75729200
H	2.36688500	-3.03489800	-2.89269200
H	2.72966300	-2.92667500	1.90758600
H	4.44135500	-2.94104000	1.39487600
H	3.27014700	-4.04152500	0.63354900
H	4.50927300	-2.75994400	-1.94863000
H	5.24602100	-1.35350200	-1.14371600
H	4.04936100	-1.10681700	-2.43525900
H	-1.78236400	0.02496200	2.55594500
H	-1.69388800	-1.14047400	3.89154600
H	-1.40183400	-1.67441900	2.21900800
H	0.72717000	-2.68951200	3.05576400
H	0.32412500	-2.12635600	4.69773900
H	1.91202300	-1.74371300	3.99616000
H	-0.07865000	1.49451900	3.74463800
H	-0.18328100	0.33578000	5.09598900
H	1.40172000	0.78546500	4.44295900
H	3.65326500	2.63355000	1.53609300
H	2.43212200	2.70876200	2.83345200
H	1.90636300	2.61459600	1.13865200

H	3.47331800	0.11521800	4.32702300
H	4.75480100	0.30120300	3.11107800
H	3.93419700	-1.26293800	3.29061800
H	-3.07867200	-1.83557400	-1.75491900
H	-4.73659400	-2.11757100	-1.14487200
H	-3.30264400	-2.45042000	-0.12808200
C	-5.08912600	1.59664400	0.32415800
H	-5.89614700	-0.18047300	-0.59965600
H	-1.77595200	2.77286300	2.24387900
H	-1.13905800	3.06946500	0.62260500
H	-0.62364300	1.64001600	1.53457400
H	-3.95702500	3.13989100	1.29233000
H	-2.57352500	4.32673200	-2.89689900
H	-1.84987200	3.42402200	-4.25929000
H	-3.61276500	3.38105300	-3.99484600
H	-3.55707800	0.76627800	-3.84758300
H	-1.79379400	0.81663400	-4.14743500
H	-2.43709000	0.02432600	-2.67129300
C	-6.41619000	2.30307700	0.42729300
H	-7.09294000	1.79005100	1.12878300
H	-6.92990900	2.34226100	-0.54533200
H	-6.28926700	3.33485300	0.78297200

Table S9

Ge=P-PC

Atomic Number	Coordinates (Angstroms) X	Y	Z
Ge	0.13444000	-0.55543700	0.16946800
C	0.59062600	1.07640200	-2.24595300
C	-3.08804500	0.30884300	0.35187600
P	-1.91479700	-0.65151900	-0.71701600
N	1.04182200	1.26440000	-1.10275600
C	1.90752200	2.42397600	-0.77854200
N	1.32648500	-1.80472300	-0.57336200
N	0.80204200	-0.05502400	1.83928900

C	2.49876500	3.05529800	-2.03191300
C	1.14118800	3.43581800	0.06166000
H	2.72270800	2.00029200	-0.17903600
N	0.24867900	0.74913300	-3.34757400
C	0.82839700	-2.99998300	-1.32603800
Si	3.02201300	-1.34521000	-0.63386900
C	-0.12449100	0.17244300	2.98790800
Si	2.54580700	0.09009900	2.01771100
C	-4.04416000	-0.39887600	1.11974000
C	-3.19494100	1.71658900	0.27712300
H	1.70691500	3.47688400	-2.67161200
H	3.17907100	3.87165500	-1.75256600
H	3.06436000	2.31778900	-2.61827900
H	0.37160500	3.94231900	-0.53979700
H	1.83212400	4.19683200	0.45283800
H	0.64762100	2.93811500	0.90747000
C	-0.97171000	0.92850500	-4.12977700
C	-0.17194300	-3.79665800	-0.47742800
C	0.21604900	-2.58854200	-2.67226500
C	1.98769800	-3.95725400	-1.63862500
C	4.27856500	-2.60417100	-0.02080200
C	3.59364800	-0.70101600	-2.30329000
O	3.11166200	-0.08463800	0.45924300
C	-0.80958200	1.53484400	2.84930000
C	-1.16715400	-0.94602500	3.05981800
C	0.63635900	0.14894800	4.32059800
C	3.15363400	1.77450400	2.59068700
C	3.33312900	-1.25560100	3.06618200
C	-4.09240800	-1.90699400	1.13963500
C	-5.00012300	0.30918400	1.85678900
C	-2.26646300	2.51166600	-0.59870700
C	-4.16680500	2.38709900	1.02290700
C	-1.14236100	2.40084900	-4.48867200
C	-0.91339000	0.03261800	-5.35682300
H	-1.79771900	0.60302700	-3.47395600
H	0.29704700	-4.07090900	0.48008600
H	-0.46004600	-4.72029400	-1.00142400
H	-1.08369900	-3.22076100	-0.27619700

H	0.96393300	-2.04791500	-3.27356100
H	-0.11004800	-3.47776000	-3.23385400
H	-0.65572400	-1.93384000	-2.53983900
H	2.45743500	-4.33635200	-0.72141300
H	1.59566400	-4.81627700	-2.20031900
H	2.75659000	-3.48491800	-2.26987300
H	3.90313700	-3.17242400	0.84244100
H	5.16277400	-2.03522300	0.30843300
H	4.60116800	-3.31600700	-0.79155200
H	3.95123300	-1.52012600	-2.94523000
H	4.43549700	-0.00654400	-2.15371700
H	2.79477700	-0.17694700	-2.84769300
H	-0.06815400	2.34424400	2.93927200
H	-1.57387100	1.67754700	3.62846400
H	-1.30965500	1.61951300	1.87553600
H	-1.80506400	-0.94723600	2.17034000
H	-1.81843600	-0.81129000	3.93619300
H	-0.66477900	-1.92279700	3.13764000
H	1.42232500	0.91665400	4.36792100
H	-0.06971900	0.36011100	5.13586000
H	1.08129600	-0.83791200	4.50725700
H	3.32679200	1.81408400	3.67530600
H	2.45665200	2.58157500	2.32413000
H	4.11517200	1.97337400	2.09191700
H	3.31161000	-1.02337600	4.13970000
H	4.38608700	-1.37185900	2.76638800
H	2.82292100	-2.21838800	2.91036500
H	-4.30457600	-2.29902100	0.13370500
H	-4.87355500	-2.26074400	1.82598600
H	-3.13011400	-2.34767600	1.43592600
C	-5.06986400	1.70206800	1.83786900
H	-5.72232300	-0.25102200	2.45728200
H	-2.49325000	3.58602700	-0.55031700
H	-2.33978800	2.17029500	-1.64339700
H	-1.22017900	2.35633600	-0.30316700
H	-4.22403400	3.47761500	0.96067300
H	-1.19978200	3.02229400	-3.58321000
H	-0.29560100	2.74646900	-5.10017100

H	-2.06763100	2.54248600	-5.06406200
H	-1.84407800	0.12294400	-5.93328600
H	-0.06954900	0.31989200	-6.00114800
H	-0.78536700	-1.01690500	-5.06090400
C	-6.08033700	2.44503200	2.67196600
H	-5.64426700	2.75013400	3.63631700
H	-6.95664800	1.81932100	2.88852800
H	-6.42343100	3.35687600	2.16385800

Table S10

Ge=As-PC

Atomic Number	Coordinates (Angstroms) X	Y	Z
Ge	0.28416900	-0.52678000	0.19217900
C	0.71988600	1.08361100	-2.20084900
C	-3.06877800	0.40676400	0.42822300
As	-1.88692100	-0.67969500	-0.72951100
N	1.14685200	1.25846200	-1.04288500
C	1.98839800	2.43455500	-0.69836400
N	1.50402200	-1.77653800	-0.51627600
N	0.91915000	-0.01950200	1.87558400
C	2.61172600	3.06240100	-1.93776100
C	1.18405800	3.44363900	0.10800800
H	2.79007900	2.02489100	-0.07300600
N	0.40746000	0.78017600	-3.31546700
C	1.03243600	-2.98261200	-1.26649500
Si	3.19445200	-1.29838500	-0.55233800
C	-0.02823700	0.19936200	3.00974100
Si	2.65738900	0.13798100	2.08580600
C	-4.00174500	-0.25108000	1.26243500
C	-3.13117300	1.81364100	0.32119100
H	1.83721900	3.47188500	-2.60583400
H	3.27322200	3.88809100	-1.64137100
H	3.20485300	2.32768700	-2.49970800
H	0.42994800	3.93842000	-0.52198700

H	1.85482300	4.21441300	0.51494100
H	0.66838700	2.94820600	0.94145300
C	-0.78586600	0.96283300	-4.13798300
C	0.04198700	-3.79275800	-0.41943600
C	0.42530500	-2.58578200	-2.61963700
C	2.20806900	-3.92336800	-1.56931100
C	4.45863200	-2.53976000	0.07984800
C	3.77918900	-0.64947300	-2.21580200
O	3.25250700	-0.03270100	0.53660100
C	-0.72955500	1.55190700	2.85618500
C	-1.05689400	-0.93264400	3.07194700
C	0.71218600	0.19169600	4.35420300
C	3.23817300	1.83040800	2.66496600
C	3.43964400	-1.20021000	3.14728600
C	-4.10885500	-1.75577600	1.32641800
C	-4.89425400	0.50751000	2.03002200
C	-2.22542100	2.56343200	-0.61847100
C	-4.04152800	2.53483100	1.09795600
C	-0.93020300	2.43476500	-4.50897800
C	-0.69558400	0.05662900	-5.35512800
H	-1.63423000	0.65052100	-3.50493300
H	0.51676800	-4.06495500	0.53566600
H	-0.23861600	-4.71758100	-0.94550000
H	-0.87665400	-3.23019400	-0.21060800
H	1.17316000	-2.04201400	-3.21836500
H	0.11039600	-3.48027900	-3.17894000
H	-0.45270000	-1.93664700	-2.50282500
H	2.67588700	-4.29500400	-0.64819700
H	1.83238200	-4.78869100	-2.13256300
H	2.97516400	-3.44112600	-2.19520300
H	4.07805800	-3.11311400	0.93743000
H	5.32983000	-1.95865800	0.42222400
H	4.80222700	-3.24693100	-0.68612500
H	4.14029600	-1.46651400	-2.85837600
H	4.61986100	0.04492200	-2.05977500
H	2.98327300	-0.12413500	-2.76352500
H	-0.00419800	2.37239700	2.97314000
H	-1.52060700	1.67889500	3.61107500

H	-1.20055300	1.63422400	1.86779900
H	-1.68227500	-0.94666300	2.17370200
H	-1.72118700	-0.80287900	3.93941600
H	-0.54283700	-1.90250800	3.15974700
H	1.48888900	0.96827000	4.40980900
H	-0.00916000	0.39983400	5.15680500
H	1.16439100	-0.78928600	4.55398000
H	3.41027200	1.86862100	3.74982800
H	2.52498400	2.62482400	2.40220200
H	4.19557200	2.04983600	2.16700300
H	3.39675000	-0.96915000	4.22038000
H	4.49883200	-1.30593300	2.86605500
H	2.94138100	-2.16763100	2.98161400
H	-4.43795600	-2.15985100	0.35731800
H	-4.83465100	-2.05962200	2.09293400
H	-3.14230400	-2.23301800	1.53716500
C	-4.92101300	1.90068200	1.97711700
H	-5.60047400	-0.01280100	2.68319400
H	-2.39263100	3.64789600	-0.55230500
H	-2.39196800	2.23377200	-1.65611000
H	-1.17075000	2.35188100	-0.39733100
H	-4.06685200	3.62486500	1.01081900
H	-1.01485300	3.05991200	-3.60822900
H	-0.06031000	2.77126100	-5.09238500
H	-1.83389800	2.58037300	-5.11663500
H	-1.60736400	0.14940500	-5.96058600
H	0.17008200	0.33148100	-5.97541700
H	-0.58599100	-0.99136500	-5.04628200
C	-5.86078600	2.69705700	2.84404700
H	-5.36868500	2.99705700	3.78269000
H	-6.75116600	2.11159000	3.11021700
H	-6.18906200	3.61508400	2.33738700

Table S11

Ge=Sb-PC

Atomic Coordinates (Angstroms)

Number	X	Y	Z
Ge	-0.23944500	-0.58332800	-0.54916800
N	-0.42228900	-0.33236100	1.61323500
C	0.63363800	-0.67207600	2.19224700
Sb	2.13765000	0.52709300	-0.99942300
C	1.54883100	2.65198600	-0.79869500
C	-1.53329600	0.32629800	2.34656000
N	-0.27046200	-2.47814800	-0.55957900
N	-1.98577400	-0.01779100	-0.95105300
C	-2.05294600	-0.57586400	3.45648000
C	-1.11937800	1.70180900	2.85301000
H	-2.31079700	0.45769300	1.58640400
N	1.61543300	-1.12560000	2.69318300
C	0.88126500	-3.24991300	-1.11546400
Si	-1.48309300	-3.27055100	0.43409000
C	-2.22386700	1.27120200	-1.66615700
Si	-3.32305100	-1.01788600	-0.40086500
C	1.25948600	3.44351600	-1.93293600
C	1.44963300	3.25804900	0.47796300
H	-1.26872600	-0.75486400	4.21016000
H	-2.90328700	-0.09465900	3.96067800
H	-2.38379600	-1.53494000	3.03938000
H	-0.31650500	1.61790400	3.60330800
H	-1.98053900	2.19081100	3.33053300
H	-0.76759000	2.34220300	2.03191700
C	2.99358900	-0.70272200	2.94195900
C	1.19483900	-2.79125400	-2.54460600
C	2.10741800	-3.12875200	-0.19906400
C	0.54583300	-4.74557200	-1.21168100
C	-2.43945000	-4.69495000	-0.33968700
C	-0.86299100	-3.84380300	2.11078900
O	-2.60831700	-2.06271900	0.68867500
C	-1.97537300	2.44645800	-0.71393000
C	-1.31992200	1.37411400	-2.89636900
C	-3.66625200	1.35982100	-2.18727200
C	-4.66270200	-0.13569400	0.59135500
C	-4.11397300	-2.02978900	-1.76976900

C	1.56329900	3.01493300	-3.35019300
C	0.73499800	4.73435400	-1.76452500
C	1.95137500	2.57980800	1.73096200
C	0.92515400	4.54752800	0.60552700
C	3.03620600	0.12929900	4.21851200
C	3.88515800	-1.93194500	3.01420600
H	3.28244100	-0.08419100	2.07521500
H	0.29968800	-2.91615700	-3.17251400
H	2.00849700	-3.39674400	-2.97091000
H	1.50465600	-1.73899600	-2.59509800
H	1.85481800	-3.50378400	0.80595700
H	2.95208700	-3.71617700	-0.59097700
H	2.44057100	-2.08658000	-0.09115600
H	-0.31612800	-4.92350200	-1.86814700
H	1.41098400	-5.27667300	-1.63235600
H	0.34364700	-5.19006600	-0.22527800
H	-2.63012200	-4.53854700	-1.41069000
H	-3.41338500	-4.73803700	0.17379100
H	-1.94729500	-5.66832600	-0.21341800
H	-0.23632500	-4.74319600	2.01162000
H	-1.72140200	-4.10869000	2.74816100
H	-0.26649000	-3.08027800	2.62970200
H	-2.67143100	2.39827100	0.13917500
H	-2.11609600	3.41232000	-1.22328600
H	-0.94491300	2.42712000	-0.32886200
H	-0.26095000	1.32621300	-2.62301900
H	-1.48993300	2.32701600	-3.42021500
H	-1.53357100	0.54438200	-3.58787600
H	-4.41168600	1.35286800	-1.38100000
H	-3.78895700	2.30585600	-2.73295000
H	-3.88203300	0.54076200	-2.88788000
H	-5.60041600	-0.05567900	0.02245800
H	-4.37915600	0.87645400	0.91469900
H	-4.86159800	-0.73839000	1.49084800
H	-4.81395500	-1.43459700	-2.37278500
H	-4.67395700	-2.87282100	-1.33649700
H	-3.34060100	-2.43314400	-2.44074700
H	2.57590300	3.35502500	-3.62168900

H	0.86133400	3.47362400	-4.06084200
H	1.54972700	1.92646300	-3.47532900
C	0.52389500	5.29190300	-0.50679400
H	0.48967600	5.32261200	-2.65395200
H	1.73976800	3.19244800	2.61953000
H	3.03916200	2.41738900	1.66835800
H	1.49801800	1.59071500	1.86307900
H	0.83512700	4.98940900	1.60240600
H	2.39891700	1.02012600	4.12844000
H	2.69701600	-0.46608700	5.07866600
H	4.06688900	0.45923800	4.40740200
H	4.92739900	-1.62383900	3.17293200
H	3.57735900	-2.58218100	3.84591300
H	3.83020700	-2.50486000	2.07940000
C	-0.10911700	6.64869900	-0.34544600
H	-1.19139200	6.55407500	-0.16315600
H	0.02272600	7.26096800	-1.24760400
H	0.32104100	7.19135000	0.50773300

Table S12

Ge=Bi-PC

Atomic Number	Coordinates (Angstroms) X	Y	Z
Bi	-1.40932800	0.14664600	-1.78952000
C	-1.30537700	-0.04470700	1.99617100
N	-0.09390500	-0.28258500	1.79451500
Ge	0.62804200	0.73931900	0.00231200
C	-0.84978700	-2.07572100	-2.11748900
C	-0.06457600	-2.47811900	-3.21885800
C	-1.24728300	-3.06200600	-1.18476100
N	2.40006800	0.12747500	0.13744000
N	0.61646600	2.49584400	0.72268900
C	0.23102500	-1.58162500	-4.39953800
C	0.41676400	-3.79509400	-3.28613400
C	-2.24962500	-2.77558200	-0.09064100

C	-0.74886700	-4.36516900	-1.28385000
C	2.93283700	-0.85937200	-0.84827000
Si	3.36779300	0.68771300	1.49299100
C	-0.23122500	3.54689200	0.08933600
Si	1.28647200	2.71958500	2.32923300
H	0.26722800	-0.52118800	-4.12466600
H	-0.56913200	-1.68975400	-5.14980300
H	1.17601100	-1.86401500	-4.88496900
C	0.11724400	-4.74430400	-2.31260800
H	1.04530300	-4.08446600	-4.13399700
H	-1.93131200	-1.93307300	0.53564300
H	-3.22146500	-2.49066100	-0.52382400
H	-2.39740700	-3.65917500	0.54756700
H	-1.05069300	-5.10984800	-0.54088400
C	2.34768100	-2.24723800	-0.56304100
C	2.59830600	-0.42521000	-2.27748000
C	4.46458500	-0.94372200	-0.77293400
C	4.61769600	2.00702000	1.02329400
C	4.21207900	-0.65117500	2.51848000
O	2.24357800	1.36556300	2.52688300
C	0.07993900	3.65465900	-1.40846300
C	0.06437600	4.92894600	0.68983600
C	-1.72031300	3.26318100	0.33476600
C	0.01240900	2.73375300	3.70840600
C	2.42791500	4.19271500	2.59216800
C	0.70386800	-6.13063700	-2.36199600
H	2.70232500	-2.99048900	-1.29374800
H	2.63754600	-2.58440700	0.44535000
H	1.24892400	-2.22726700	-0.62670100
H	3.00967900	-1.14385700	-3.00247800
H	3.02827900	0.56840600	-2.47811900
H	1.51670000	-0.37014000	-2.44043600
H	4.82068800	-1.65541700	-1.53088700
H	4.82284300	-1.30271300	0.20109400
H	4.92203100	0.03214200	-0.98986100
H	4.16756600	2.72261200	0.31872900
H	4.93959000	2.55677400	1.92119700
H	5.51278700	1.57863900	0.55067500

H	3.84290400	-1.66587700	2.30987700
H	4.01851900	-0.42876300	3.57911600
H	5.30104900	-0.65204400	2.36526900
H	1.14828500	3.88038900	-1.54479000
H	-0.50984900	4.46252900	-1.86666900
H	-0.14363200	2.72934500	-1.95719800
H	-0.56743900	5.67758600	0.19168400
H	-0.17040800	4.97171900	1.76435400
H	1.11432400	5.21460300	0.54205700
H	-2.35218800	4.04663700	-0.11149100
H	-1.91643400	3.22799300	1.41846700
H	-2.02909200	2.29499900	-0.08615300
H	-0.55149900	3.67909700	3.71161600
H	0.52184800	2.65066800	4.68144900
H	-0.71509400	1.91390400	3.62551300
H	3.05132800	4.40217400	1.71159000
H	3.09887600	3.92889900	3.42541700
H	1.89434400	5.11250000	2.86629300
H	1.60945600	-6.19308000	-1.73777600
H	-0.00674100	-6.87919200	-1.98491200
H	0.98782200	-6.40799700	-3.38608000
C	0.63893800	-1.29334000	2.60054900
C	0.08768800	-2.69240200	2.35919700
C	0.63809000	-0.90939200	4.07318400
H	1.66172800	-1.25102900	2.21069300
N	-2.42128300	0.31618800	2.21091700
H	0.69256000	-3.42538000	2.91203500
H	-0.95184700	-2.77033700	2.71705700
H	0.11288200	-2.95538000	1.29238600
H	1.21933600	-1.64378400	4.64915000
H	-0.38960700	-0.90263800	4.47182700
H	1.08789000	0.08216100	4.20594800
C	-3.76747200	-0.00320600	1.73637600
C	-4.64984400	1.22593500	1.88407100
C	-4.29642700	-1.20304600	2.51350000
H	-3.66264700	-0.25876400	0.66826500
H	-5.65765400	1.00548600	1.50695700
H	-4.72380300	1.52196500	2.94054300

H	-4.23998800	2.06736200	1.31017200
H	-3.65024300	-2.08015800	2.36869600
H	-4.35083700	-0.97387100	3.58768900
H	-5.30524300	-1.45454200	2.15864700

Table S13

Ge=N-TS

Atomic Number	Coordinates (Angstroms) X	Y	Z
Ge	1.47974900	0.81387400	-1.03108400
C	0.82277000	2.76319700	-2.65140400
C	-1.38185100	1.53278000	-1.11253000
N	-0.19573300	0.98000600	-1.51588500
N	1.96220100	2.41847500	-2.14939800
C	3.18736000	2.88735600	-2.85488000
N	2.49721200	-0.49232300	-1.88825800
N	2.12855600	0.98507700	0.71139600
C	3.30533000	2.20347000	-4.21208000
C	3.20599300	4.40671900	-2.97458900
H	4.01409600	2.56005100	-2.21219300
N	-0.02098900	3.39886100	-3.18936900
C	1.83192500	-1.55078600	-2.69526500
Si	4.18247800	-0.51744000	-1.40391600
C	1.35932200	0.59168800	1.92469600
Si	3.77581100	1.58344600	0.76186000
C	-2.58754500	0.85102400	-1.46855600
C	-1.52230400	2.79685500	-0.46633800
H	2.47799300	2.51063900	-4.87262300
H	4.25247100	2.47316900	-4.70120100
H	3.26277600	1.11350600	-4.08598500
H	2.37860000	4.76240000	-3.60698600
H	4.14977700	4.73170500	-3.43466700
H	3.11892600	4.88556600	-1.98978000
C	-1.28549000	3.39469000	-3.89592600
C	0.88706700	-2.35623200	-1.79344400

C	1.04406800	-0.93018300	-3.85642800
C	2.86800600	-2.50485300	-3.29941600
C	4.58651300	-1.83764300	-0.13003800
C	5.46209000	-0.57768000	-2.77108000
O	4.38838000	0.98798300	-0.68165100
C	-0.11907500	0.33112200	1.62471000
C	1.93899100	-0.70734900	2.50389400
C	1.43750000	1.71438700	2.96874700
C	3.83311600	3.45030700	0.67319200
C	4.89994000	0.99240500	2.14230600
C	-2.49712200	-0.54064100	-2.03113900
C	-3.82726100	1.45244400	-1.28418100
C	-0.33434600	3.58332800	0.03273200
C	-2.79516300	3.35295000	-0.28691000
C	-1.34539700	4.59673800	-4.82995600
C	-1.48343500	2.06656700	-4.61465000
H	-2.05552400	3.49769600	-3.11268700
H	1.45058700	-2.79694800	-0.95600600
H	0.39487700	-3.16728300	-2.35175500
H	0.10482700	-1.69549800	-1.39202100
H	1.72631700	-0.41274600	-4.54747400
H	0.51389900	-1.71302100	-4.42063000
H	0.30827500	-0.21107000	-3.47316400
H	3.47991000	-2.99423800	-2.52614900
H	2.35036100	-3.29713600	-3.85826000
H	3.53141600	-1.97750100	-3.99922600
H	3.90641300	-1.75475200	0.73061500
H	5.62064300	-1.71751900	0.22913000
H	4.48998000	-2.85451000	-0.53743400
H	5.65777900	-1.59890500	-3.12492700
H	6.39791100	-0.17745500	-2.35101700
H	5.18637800	0.04510200	-3.63345500
H	-0.63896900	1.23126400	1.27665500
H	-0.61347800	-0.00988000	2.54537200
H	-0.24925000	-0.45332900	0.86333000
H	1.84775300	-1.51526600	1.76176500
H	1.38992400	-1.00837700	3.40872700
H	2.99822700	-0.59858300	2.77131600

H	1.01168900	2.64245300	2.56106300
H	0.87491200	1.44231900	3.87383900
H	2.47575800	1.91395600	3.27380300
H	4.83116000	3.81428000	0.38584900
H	3.57192800	3.89152700	1.64713000
H	3.09726800	3.80520700	-0.06328200
H	4.57262800	1.28867100	3.14826500
H	5.88207700	1.45738900	1.96087500
H	5.03897400	-0.09688600	2.12430000
H	-1.80944900	-0.59579200	-2.88760200
H	-3.48521600	-0.90883200	-2.33868000
H	-2.09278000	-1.23293600	-1.27543000
C	-3.96314800	2.72166000	-0.70723000
H	-4.72592700	0.90737400	-1.59040100
H	-0.62653600	4.20668200	0.89023300
H	0.06720300	4.26435800	-0.73640300
H	0.49484900	2.94046600	0.35900400
H	-2.86885500	4.32929500	0.20287200
H	-1.19701800	5.53236400	-4.27498600
H	-0.56867000	4.51971200	-5.60516900
H	-2.32633300	4.63206600	-5.32215500
H	-2.47905400	2.03960800	-5.07904700
H	-0.72189900	1.93605700	-5.39919500
H	-1.40395800	1.24252000	-3.89431900
C	-5.32130600	3.34290400	-0.50671900
H	-5.91343700	2.78288800	0.23402500
H	-5.90050800	3.35341600	-1.44227800
H	-5.23541500	4.37856900	-0.15069100

Table S14

Ge=P-TS

Atomic Number	Coordinates (Angstroms) X	Y	Z
Ge	1.36275500	0.64695700	-1.14244800
C	1.30902000	2.16201400	-3.39309900

C	-1.94132500	1.46345300	-1.04964100
P	-0.70617800	0.57737700	-2.11992400
N	2.02565400	2.22187200	-2.33560100
C	2.98494600	3.35314600	-2.15223000
N	2.49385200	-0.73581700	-1.75223100
N	1.94993300	1.11491600	0.57135900
C	3.48040800	3.91877000	-3.47692900
C	2.36559700	4.43809700	-1.28004800
H	3.83256900	2.90016300	-1.62408200
N	1.00105800	2.18641600	-4.55172400
C	1.96482100	-1.92941700	-2.47478800
Si	4.19516800	-0.31568900	-1.80263600
C	0.97905300	1.30141100	1.68598100
Si	3.68857000	1.19818700	0.80324100
C	-2.90422600	0.68365300	-0.36263600
C	-2.08096800	2.87078000	-1.02565000
H	2.66411000	4.39323300	-4.04214100
H	4.24242400	4.68480600	-3.27619700
H	3.93175500	3.14118700	-4.10715500
H	1.57435200	4.97462800	-1.82491300
H	3.13426600	5.16853800	-0.98715800
H	1.92869200	4.00084400	-0.37156200
C	-0.23133200	2.28524100	-5.30425700
C	0.88470600	-2.62785000	-1.63808600
C	1.42933800	-1.54097200	-3.86133200
C	3.08237500	-2.95851700	-2.69557400
C	5.43138900	-1.57440100	-1.15511600
C	4.75776400	0.29828200	-3.48344800
O	4.27705100	0.97934300	-0.74550200
C	0.29187400	2.66303300	1.55468800
C	-0.05984200	0.17612000	1.67862400
C	1.68409200	1.24494500	3.04738700
C	4.35978900	2.86021300	1.35800100
C	4.40607900	-0.15837300	1.88727500
C	-2.90029900	-0.82449800	-0.41779500
C	-3.90891700	1.31646600	0.37876500
C	-1.13131800	3.75438000	-1.78566100
C	-3.10165000	3.46448100	-0.28045100

C	-0.45136200	3.72516000	-5.75612100
C	-0.18919700	1.30768900	-6.47034500
H	-1.02801700	1.97865000	-4.59759600
H	1.30011800	-2.89574500	-0.65452400
H	0.55151700	-3.54903800	-2.13952300
H	0.00749200	-1.98384400	-1.49556200
H	2.22127200	-1.04302800	-4.44324200
H	1.10039500	-2.43398400	-4.41509400
H	0.57133800	-0.86041500	-3.78456500
H	3.51133300	-3.29656700	-1.74255400
H	2.66612100	-3.83397900	-3.21297300
H	3.88903200	-2.56153100	-3.33190300
H	5.04674900	-2.11402700	-0.27792500
H	6.32549400	-1.01210800	-0.84168900
H	5.74046500	-2.31092000	-1.90822600
H	4.95121300	-0.53554000	-4.17505700
H	5.68665500	0.88185500	-3.38967300
H	3.98297900	0.93550000	-3.93476900
H	1.02967100	3.47286100	1.67000800
H	-0.49076400	2.79245900	2.31786900
H	-0.18632300	2.75745400	0.57109500
H	-0.66015700	0.20062700	0.76248800
H	-0.75117700	0.27950200	2.52832300
H	0.44427700	-0.80039400	1.74796700
H	2.47127300	2.00754100	3.14024400
H	0.94802700	1.44159200	3.83945400
H	2.11899200	0.25293100	3.22981100
H	4.27108500	3.02460400	2.44068000
H	3.86376200	3.69375600	0.84198400
H	5.43045800	2.89026600	1.10178700
H	4.37131400	0.09130200	2.95661100
H	5.46031700	-0.31465200	1.61092900
H	3.86564500	-1.10508200	1.73422800
H	-2.99676900	-1.17644400	-1.45501300
H	-3.72829600	-1.23437900	0.17619300
H	-1.95629200	-1.24664200	-0.04349000
C	-4.02048300	2.70435100	0.44535300
H	-4.63474700	0.69892900	0.91505300

H	-1.36931500	4.81703100	-1.63884000
H	-1.16356800	3.52447100	-2.86170800
H	-0.09662700	3.57837300	-1.46306500
H	-3.18258500	4.55524400	-0.26795900
H	-0.47786000	4.41026500	-4.89715300
H	0.35576000	4.04247500	-6.43236200
H	-1.40656700	3.80453000	-6.29299800
H	-1.13761200	1.34423800	-7.02357800
H	0.63092300	1.56279100	-7.15707400
H	-0.03645200	0.28353300	-6.10485900
C	-5.08711600	3.36708600	1.27693500
H	-4.67430200	3.72067700	2.23476900
H	-5.90591100	2.67151600	1.50406200
H	-5.50908500	4.24063800	0.76038800

Table S15

Ge=As-TS

Atomic Number	Coordinates (Angstroms) X	Y	Z
Ge	1.36962400	0.58553300	-1.16945300
C	1.31780200	2.11743300	-3.43265200
C	-2.05735900	1.48305900	-1.02369400
As	-0.80956400	0.47262200	-2.18763500
N	2.01423700	2.16862900	-2.36099200
C	2.95487300	3.31451400	-2.15611400
N	2.53517100	-0.78297600	-1.75054800
N	1.93525900	1.08007000	0.54521700
C	3.50405900	3.86087400	-3.46775600
C	2.28893900	4.40870900	-1.33196400
H	3.78322100	2.87886000	-1.58577800
N	1.03046700	2.16035200	-4.59546800
C	2.03383700	-1.99369500	-2.46312200
Si	4.23078900	-0.33833200	-1.78520900
C	0.95106500	1.28370600	1.64563900
Si	3.66885600	1.19660300	0.79633700

C	-3.00327500	0.75067800	-0.26719800
C	-2.15248300	2.89123600	-1.03983000
H	2.71125800	4.32129300	-4.07620400
H	4.25252200	4.63459300	-3.24645700
H	3.98715500	3.07557200	-4.06378700
H	1.51189800	4.92283400	-1.91757600
H	3.03673900	5.15569800	-1.02701700
H	1.82381900	3.98585600	-0.43101600
C	-0.17994000	2.29009400	-5.37694800
C	0.96534200	-2.70599100	-1.62320600
C	1.50200800	-1.62933700	-3.85740400
C	3.17112800	-3.00443700	-2.66910800
C	5.47697700	-1.57207200	-1.10937600
C	4.80217700	0.26391600	-3.46741100
O	4.28200400	0.96841400	-0.74188500
C	0.26223200	2.64112300	1.48226000
C	-0.08497400	0.15583500	1.64895500
C	1.64173300	1.25308500	3.01528000
C	4.29830000	2.88084700	1.33550000
C	4.39818200	-0.13235100	1.90589600
C	-3.05274500	-0.75845300	-0.27467700
C	-3.95234500	1.43562500	0.50044700
C	-1.21686800	3.72813600	-1.86915300
C	-3.12010100	3.53821300	-0.26565100
C	-0.38056200	3.74600500	-5.78477200
C	-0.11264100	1.35106600	-6.57288500
H	-0.99661200	1.96646300	-4.70072100
H	1.38656900	-2.96254200	-0.63923500
H	0.64633000	-3.63459700	-2.12007700
H	0.07555000	-2.07970500	-1.47855300
H	2.29481000	-1.13700300	-4.44270200
H	1.17859900	-2.53129300	-4.39943500
H	0.64180100	-0.94979400	-3.80163200
H	3.59857900	-3.32921700	-1.71092000
H	2.77370600	-3.89022400	-3.18376800
H	3.97553400	-2.59772100	-3.30201400
H	5.08925300	-2.10767600	-0.23105100
H	6.35873800	-0.99336400	-0.79095700

H	5.80612300	-2.31206500	-1.85046300
H	5.02516500	-0.57490600	-4.14390100
H	5.71545500	0.87101500	-3.36991000
H	4.01867300	0.87567900	-3.93848500
H	0.99323100	3.45515700	1.61068300
H	-0.54264600	2.77499000	2.22122700
H	-0.18762600	2.72419100	0.48417700
H	-0.67893400	0.16357100	0.72861800
H	-0.78212800	0.27193300	2.49231200
H	0.42086400	-0.81823700	1.73749400
H	2.42507800	2.02015300	3.10415400
H	0.89631900	1.46039300	3.79583400
H	2.07810000	0.26571100	3.21873600
H	4.24194300	3.03578200	2.42181500
H	3.75192300	3.69746800	0.84323000
H	5.35719000	2.95320000	1.04165600
H	4.34072100	0.12731800	2.97184000
H	5.45973300	-0.26940300	1.64792000
H	3.87989300	-1.09159900	1.75440800
H	-3.26504200	-1.13615700	-1.28536900
H	-3.83326400	-1.12156500	0.40756600
H	-2.09147600	-1.20439700	0.01706000
C	-4.02205300	2.82843400	0.52706500
H	-4.66849600	0.85674800	1.09047300
H	-1.40969600	4.80088500	-1.72831700
H	-1.31905300	3.48228500	-2.93716500
H	-0.17248600	3.52049500	-1.60119900
H	-3.17003000	4.63060600	-0.28411600
H	-0.42122400	4.40094600	-4.90308300
H	0.44281100	4.08054600	-6.43259000
H	-1.32347400	3.85028600	-6.33888300
H	-1.04535400	1.41304800	-7.14995900
H	0.72755600	1.62182700	-7.22861500
H	0.02262000	0.31410600	-6.23759100
C	-5.02770100	3.54068200	1.39314800
H	-4.62655400	3.70362600	2.40590800
H	-5.95147200	2.95501600	1.49567700
H	-5.28586000	4.52446200	0.97822200

Table S16

Ge=Sb-TS

Atomic Number	Coordinates (Angstroms) X	Y	Z
Ge	0.00000000	0.00000000	0.00000000
N	0.00000000	0.00000000	2.08341700
C	1.21228600	0.00000000	2.49247000
Sb	2.38796500	1.16204200	-0.19616000
C	1.87548900	3.29951900	-0.38452500
C	-1.09066000	0.14222600	3.10195700
N	-0.02483200	-1.85828900	-0.35705500
N	-1.71508400	0.63509300	-0.41268500
C	-0.75700700	-0.55233700	4.41607100
C	-1.43136300	1.61021200	3.31660600
H	-1.94450500	-0.37274200	2.64973100
N	2.17422200	-0.26181200	3.15927400
C	1.12492600	-2.52760100	-1.02725800
Si	-1.24326200	-2.77400600	0.50833700
C	-1.89061900	1.92042000	-1.14741000
Si	-3.07339700	-0.37002800	0.05842400
C	1.99501200	3.92213500	-1.64672000
C	1.53577400	4.09738100	0.73085900
H	0.08972500	-0.06916400	4.92590000
H	-1.62998000	-0.49351900	5.08118800
H	-0.51293700	-1.61140400	4.26266500
H	-0.61328700	2.13018800	3.83847100
H	-2.33853600	1.69781800	3.93259800
H	-1.60657300	2.11245100	2.35599200
C	3.45818000	0.33972300	3.43678300
C	1.41605700	-1.86193600	-2.37862800
C	2.36249800	-2.52798400	-0.11764300
C	0.79563000	-3.99644900	-1.32994600
C	-2.21908100	-4.05775900	-0.45731800
C	-0.59855800	-3.54685100	2.09202400

O	-2.34841400	-1.59488900	0.93636200
C	-1.65734200	3.10003600	-0.19924800
C	-0.92666500	1.99414300	-2.33568400
C	-3.30805200	2.03502700	-1.72354700
C	-4.29073500	0.43554400	1.24172800
C	-4.00177700	-1.16609600	-1.36708300
C	2.46288200	3.18951700	-2.88331300
C	1.70413500	5.28825500	-1.77506900
C	1.43714800	3.51443600	2.11716200
C	1.26501400	5.45778800	0.56547300
C	3.35792800	1.22685500	4.67373700
C	4.51229500	-0.74859700	3.57713100
H	3.68086000	0.96135500	2.54428000
H	0.51302700	-1.90410700	-3.00615400
H	2.22987500	-2.38888700	-2.89905500
H	1.71833600	-0.81219100	-2.26825900
H	2.13341300	-3.05247200	0.82339000
H	3.20905000	-3.03618700	-0.60379100
H	2.69103900	-1.51164900	0.13727300
H	-0.07134100	-4.08292800	-1.99850200
H	1.65807300	-4.46223100	-1.82680400
H	0.60042200	-4.57352400	-0.41253600
H	-2.43601700	-3.72182300	-1.48153300
H	-3.17966100	-4.19045800	0.06558600
H	-1.72341600	-5.03591800	-0.50979300
H	-0.07441900	-4.49453600	1.89682100
H	-1.42939100	-3.75762400	2.78299400
H	0.11012200	-2.86715600	2.58792000
H	-2.45297800	3.13906200	0.56164000
H	-1.64478300	4.05806500	-0.74191700
H	-0.68889900	2.99809800	0.30869200
H	0.11619500	2.01170500	-2.00031400
H	-1.09914000	2.91210200	-2.91759800
H	-1.07259500	1.12268600	-2.99280000
H	-4.08070000	1.97343500	-0.94297900
H	-3.41815800	3.01423500	-2.21040600
H	-3.49426300	1.25908800	-2.47882500
H	-5.11118100	0.95317700	0.72535900

H	-3.79519300	1.15668700	1.90769500
H	-4.73393000	-0.35696600	1.86472100
H	-4.77085600	-0.50722800	-1.79286100
H	-4.50001000	-2.07884600	-1.00528500
H	-3.30459100	-1.44904700	-2.17054400
H	3.49940900	2.84171100	-2.76231900
H	2.42091700	3.84881200	-3.76089400
H	1.86431400	2.29142800	-3.08847200
C	1.32773000	6.07210100	-0.68696000
H	1.78578200	5.75413000	-2.76107700
H	1.08914900	4.26485200	2.84110100
H	2.41335500	3.12776600	2.44781800
H	0.74206300	2.66337500	2.13080000
H	0.99362700	6.05631500	1.44005400
H	2.58393500	1.99622400	4.54272600
H	3.11044000	0.62554300	5.56085400
H	4.31960800	1.72781600	4.85104900
H	5.49868600	-0.29500700	3.74445500
H	4.27758400	-1.40648300	4.42643100
H	4.55835900	-1.35730300	2.66407000
C	0.99493900	7.53256400	-0.84593500
H	-0.08539300	7.70480200	-0.72237400
H	1.28268200	7.90182800	-1.83907000
H	1.51077000	8.14132100	-0.08948700

Table S17

Ge=Bi-TS

Atomic Number	Coordinates (Angstroms) X	Y	Z
Bi	0.00000000	0.00000000	0.00000000
C	0.00000000	0.00000000	3.18975200
N	1.27451900	0.00000000	3.31739500
Ge	2.14297000	0.73085700	1.57611400
C	0.66031500	-2.09254000	-0.70956400
C	1.12929300	-2.24858900	-2.02986300

C	0.54523900	-3.23737200	0.10737300
N	3.89102300	0.05512600	1.66770700
N	2.27285400	2.57929500	1.97419300
C	1.18871100	-1.10655800	-3.01896900
C	1.53341300	-3.51649600	-2.47601300
C	0.01338100	-3.15060700	1.51569000
C	0.94550700	-4.48633300	-0.37424400
C	4.37096100	-0.91878600	0.64583100
Si	4.91638900	0.64919800	2.95962900
C	1.47208100	3.57086000	1.20707800
Si	3.01380700	2.97614500	3.51062300
H	1.75221900	-0.24705300	-2.63048500
H	0.17903600	-0.73295600	-3.24628900
H	1.65139600	-1.43277900	-3.96037100
C	1.45874600	-4.64538300	-1.66371700
H	1.91060700	-3.62196900	-3.49726100
H	0.59047400	-2.42219500	2.10340400
H	-1.03015700	-2.80026700	1.51800300
H	0.06012200	-4.12563100	2.02139000
H	0.85939800	-5.36159800	0.27674300
C	3.78732700	-2.30560200	0.93041200
C	3.97682100	-0.46026600	-0.76236100
C	5.90150500	-1.02185200	0.66272600
C	6.33153800	1.75194000	2.40292700
C	5.55766400	-0.66379600	4.14111000
O	3.87236500	1.58780600	3.86956200
C	1.77499400	3.45459300	-0.29260300
C	1.84363400	5.00342300	1.61581800
C	-0.02762100	3.40069500	1.49368800
C	1.76496200	3.23746000	4.88653100
C	4.26663600	4.37709800	3.53214800
C	1.90641400	-5.99902000	-2.15056100
H	4.00577600	-3.01183000	0.11418500
H	4.20530600	-2.71036500	1.86580200
H	2.69462300	-2.24768900	1.02832800
H	4.40083500	-1.13585300	-1.52057500
H	4.34954200	0.55972600	-0.94495200
H	2.88869600	-0.46751600	-0.89247100

H	6.21739400	-1.78725000	-0.06007700
H	6.28631500	-1.32788900	1.64675600
H	6.36710500	-0.06974100	0.37271900
H	6.01610100	2.38611800	1.56030100
H	6.62669000	2.40736300	3.23695200
H	7.21703300	1.18046100	2.09264900
H	4.84332900	-1.49210400	4.25469700
H	5.69743800	-0.19471300	5.12750400
H	6.52421700	-1.08462300	3.83062600
H	2.85273800	3.60260800	-0.45782300
H	1.22412300	4.22095400	-0.85841600
H	1.49231300	2.47450000	-0.69978900
H	1.24035900	5.71309500	1.03249500
H	1.63287400	5.19637500	2.67930800
H	2.90344700	5.21251000	1.41787500
H	-0.62434900	4.13784000	0.93539800
H	-0.22002700	3.53640600	2.56950100
H	-0.39225400	2.40146300	1.21852700
H	1.34522300	4.25448800	4.86832600
H	2.23718200	3.08515100	5.86930100
H	0.93129000	2.52749600	4.78160000
H	4.88637900	4.38501800	2.62394200
H	4.93123800	4.20354200	4.39346600
H	3.81045700	5.36877800	3.65052700
H	2.75206100	-6.37297400	-1.55377500
H	1.09592100	-6.73783900	-2.06512600
H	2.22358200	-5.96050200	-3.20094600
C	1.85872600	-0.61305900	4.55497800
C	2.11316600	-2.09856800	4.34110700
C	1.00569200	-0.36569800	5.79268000
H	2.81136800	-0.08864700	4.68619100
N	-1.15324900	0.11473800	3.50662500
H	2.68740100	-2.50666600	5.18599700
H	1.16364000	-2.65204300	4.27525600
H	2.67863400	-2.26622400	3.41509900
H	1.53834100	-0.75135500	6.67335500
H	0.03805000	-0.88482700	5.72817600
H	0.81710200	0.70441200	5.94715900

C	-2.42038600	-0.39902500	3.04437600
C	-3.49061900	0.67345000	3.18725900
C	-2.76682100	-1.68003000	3.79723000
H	-2.26259200	-0.62916900	1.96905600
H	-4.44808200	0.30318000	2.79613300
H	-3.62332300	0.94691600	4.24401900
H	-3.20849000	1.57454900	2.62603500
H	-1.97037200	-2.42870900	3.68249900
H	-2.90383400	-1.47238700	4.86865500
H	-3.70129900	-2.10297200	3.40320800

Table S18

Ge=N-Prod

Atomic	Coordinates (Angstroms)		
Number	X	Y	Z
Ge	0.03021700	0.08021800	-0.06626300
N	-0.07212900	-0.08099100	1.79832800
N	1.77822900	-0.20048800	0.58354100
C	-0.96676300	0.00888200	2.94981700
C	1.31889200	-0.21878800	1.92374500
N	-0.25893200	1.73757400	-0.83334700
N	-0.60391300	-1.23704800	-1.20501600
C	2.82816700	-1.03419500	0.12374100
C	-0.90369900	-1.25550800	3.81229600
C	-0.71983000	1.25199200	3.81073600
H	-1.98721000	0.07661800	2.53887400
N	1.94704900	-0.27977700	3.02490400
C	-0.64729100	2.90965100	-0.01530800
Si	0.27271500	1.80282500	-2.50617800
C	-1.81851200	-2.05485000	-0.96265900
Si	0.28044700	-1.25494400	-2.73425300
C	3.87532900	-0.46020600	-0.62292400
C	2.82156800	-2.41864100	0.38245300
H	0.08807500	-1.33631100	4.27445200
H	-1.66621400	-1.20908300	4.60344500

H	-1.08496600	-2.15712100	3.21144900
H	0.31126200	1.23897100	4.18759800
H	-1.40993300	1.25632300	4.66712000
H	-0.87811400	2.17766800	3.24181500
C	3.39561300	-0.19745700	3.08667700
C	-1.97051900	2.60326600	0.69673000
C	0.46776600	3.25116300	0.98306400
C	-0.89107800	4.13316100	-0.90742500
C	-1.14453700	1.99483900	-3.72079100
C	1.60424500	3.06201400	-2.90443200
O	0.96200300	0.28036000	-2.68962700
C	-2.19659600	-2.05113100	0.51698200
C	-3.00974800	-1.48521500	-1.74819600
C	-1.55417000	-3.50513400	-1.38702600
C	1.65338000	-2.51432400	-2.84185500
C	-0.76223800	-1.40927200	-4.28980300
C	3.84850000	1.00894200	-0.92882400
C	4.91061400	-1.27579000	-1.07821700
C	1.66233600	-3.06452700	1.09254000
C	3.89017500	-3.19618100	-0.07574300
C	3.75699500	0.96825200	4.00706100
C	3.98482200	-1.50510900	3.61090400
H	3.83580400	0.00806500	2.09458500
H	-2.75880400	2.42858600	-0.05089600
H	-2.27869800	3.43725900	1.34520400
H	-1.88656100	1.70702200	1.32450300
H	1.38840700	3.50225300	0.43512100
H	0.18888700	4.11176800	1.61056700
H	0.68917000	2.40280500	1.64556400
H	-1.66852500	3.92503500	-1.65716100
H	-1.23679100	4.97420400	-0.28997800
H	0.02655500	4.45610400	-1.41775900
H	-2.00352200	1.37782200	-3.41750100
H	-0.82988800	1.68792400	-4.73017600
H	-1.48036400	3.04034600	-3.77975700
H	1.17935300	3.98025700	-3.33436000
H	2.28059000	2.61887600	-3.65152300
H	2.20311400	3.33128000	-2.02309800

H	-1.36532300	-2.39790400	1.14343100
H	-3.06497600	-2.70366100	0.68423200
H	-2.47728600	-1.03796500	0.84607000
H	-3.15552400	-0.42560300	-1.48624000
H	-3.93352700	-2.03320200	-1.50825500
H	-2.84904600	-1.55342800	-2.83130300
H	-0.72792500	-3.93174800	-0.79982100
H	-2.45069400	-4.12247900	-1.23057600
H	-1.28981500	-3.57326800	-2.45328600
H	1.89229500	-2.72974000	-3.89417400
H	1.36758300	-3.45791200	-2.35375300
H	2.56282100	-2.14284800	-2.34871600
H	-1.18054100	-2.41858600	-4.41801600
H	-0.08172700	-1.22911200	-5.13780000
H	-1.57990100	-0.68111300	-4.35338600
H	3.06907800	1.21139800	-1.67710800
H	4.81185300	1.35037100	-1.32946400
H	3.59622500	1.59005000	-0.02961100
C	4.94519600	-2.64728300	-0.80437800
H	5.72077800	-0.82462200	-1.65780500
H	1.59512100	-2.74734400	2.14513900
H	1.74876600	-4.15867800	1.06291800
H	0.71894900	-2.77526900	0.60475500
H	3.88252200	-4.27052400	0.12760100
H	3.33319100	1.90656600	3.62029300
H	3.34037100	0.79440100	5.01077200
H	4.84788900	1.08388200	4.09112900
H	5.07266700	-1.41616300	3.74763100
H	3.52746000	-1.75493500	4.58034700
H	3.79696900	-2.33223900	2.91201500
C	6.09603400	-3.49638200	-1.27750700
H	6.99200400	-3.32075200	-0.66228400
H	6.36176700	-3.26151900	-2.31788700
H	5.85349500	-4.56552800	-1.21743500

Table S19

Ge=P-Prod

Atomic Number	Coordinates (Angstroms) X	Y	Z
Ge	0.06004000	0.18439300	-0.09301500
N	0.09448800	0.04373900	1.80359100
P	2.31074300	0.44309200	0.39787700
C	-0.85729100	-0.06268500	2.92209200
C	1.46857200	0.10494600	2.05558300
N	-0.58032400	1.75908100	-0.87323700
N	-0.51785400	-1.20111000	-1.19488400
C	3.25554300	-1.08119000	-0.05402000
C	-0.65467400	-1.33461500	3.75262200
C	-0.84699300	1.17671200	3.82080000
H	-1.85452800	-0.13269200	2.46557400
N	1.99481300	0.03282800	3.20772700
C	-1.18352900	2.85562000	-0.09011700
Si	0.23103300	1.99268000	-2.41890300
C	-1.69081000	-2.07259300	-0.95663700
Si	0.28083100	-1.08015800	-2.76624700
C	4.32265100	-0.85408500	-0.95798900
C	3.00449900	-2.39832600	0.39671600
H	0.26669400	-1.26036200	4.34167300
H	-1.50750900	-1.46746900	4.43460100
H	-0.58502300	-2.22591600	3.11386900
H	0.14516500	1.30577800	4.27130900
H	-1.58670100	1.05677400	4.62610800
H	-1.10186700	2.08392800	3.25614300
C	3.42852600	0.17298300	3.37311800
C	-2.39379300	2.30932500	0.67358600
C	-0.15528300	3.46208000	0.87692300
C	-1.69102500	3.95937900	-1.02620600
C	-0.92706500	2.40410800	-3.83592500
C	1.67096100	3.18600100	-2.36872200
O	0.90662300	0.47670000	-2.69149000
C	-2.06147600	-2.06703100	0.52317600
C	-2.92073600	-1.57207400	-1.73261200
C	-1.34895500	-3.50629900	-1.37915300

C	1.68295600	-2.28058300	-3.03002700
C	-0.83334200	-1.17471600	-4.27571700
C	4.56741600	0.50293400	-1.57243000
C	5.13780700	-1.91898300	-1.34614500
C	1.81087200	-2.77138600	1.23634000
C	3.86062300	-3.42878100	-0.00824600
C	3.69509900	1.29413500	4.37418100
C	4.03526500	-1.14741500	3.84332100
H	3.91168900	0.44752800	2.41255200
H	-3.13926200	1.92203400	-0.03674900
H	-2.86438600	3.08939900	1.29073600
H	-2.09613900	1.49203000	1.34396100
H	0.69092300	3.88213700	0.31258600
H	-0.60160800	4.26479600	1.48439700
H	0.24194100	2.69660900	1.55958800
H	-2.42220000	3.55821700	-1.74263200
H	-2.17852300	4.75078000	-0.43918900
H	-0.86721500	4.42879700	-1.58578400
H	-1.89185300	1.88868900	-3.72013600
H	-0.46932300	2.07537100	-4.78189900
H	-1.12179100	3.48329800	-3.91127000
H	1.35687900	4.23130500	-2.23436700
H	2.23608200	3.12004800	-3.31134600
H	2.34314200	2.91415700	-1.54049200
H	-1.20497400	-2.33564300	1.15387200
H	-2.87874800	-2.77540700	0.71824500
H	-2.41778300	-1.06675700	0.81577400
H	-3.08470700	-0.50496400	-1.51728400
H	-3.81991100	-2.13308300	-1.43556400
H	-2.79700400	-1.68910700	-2.81555300
H	-0.47833700	-3.87255000	-0.81521400
H	-2.19933700	-4.18066500	-1.19993600
H	-1.10849000	-3.55749400	-2.45270800
H	2.25601500	-1.98428500	-3.92224800
H	1.29281200	-3.29554300	-3.20042600
H	2.36912900	-2.31662700	-2.17388500
H	-1.26251700	-2.17479800	-4.43487100
H	-0.19976400	-0.94538200	-5.14768000

H	-1.65098200	-0.44226300	-4.24906900
H	3.68370500	0.82917500	-2.14374800
H	5.42639200	0.46662300	-2.25470400
H	4.75729600	1.26896300	-0.80660900
C	4.93750600	-3.21395300	-0.86747600
H	5.95102900	-1.72937100	-2.05163200
H	1.84985400	-2.33675700	2.24493600
H	1.74300900	-3.86279400	1.33406000
H	0.88867500	-2.41423700	0.75688100
H	3.65906100	-4.44282100	0.34689600
H	3.26013800	2.23818200	4.01632100
H	3.23251100	1.04814900	5.34184900
H	4.77466400	1.43962700	4.52537600
H	5.10735400	-1.02661900	4.05684300
H	3.53021800	-1.48664500	4.76040300
H	3.92833700	-1.92606900	3.07429600
C	5.85569000	-4.33894500	-1.26376600
H	6.73707800	-4.37086900	-0.60444500
H	6.21824500	-4.21275400	-2.29297200
H	5.35051900	-5.31115500	-1.18987800

Table S20

Ge=As-Prod

Atomic Number	Coordinates (Angstroms) X	Y	Z
Ge	0.05430800	0.21441900	-0.12170700
N	0.11028200	0.08345500	1.77946000
As	2.40956400	0.54299700	0.31495500
C	-0.84967800	-0.04525300	2.89243000
C	1.47609800	0.16255100	2.05998100
N	-0.63034200	1.78047200	-0.88814700
N	-0.53918800	-1.17583500	-1.21489800
C	3.40411700	-1.10911600	-0.09112900
C	-0.64148600	-1.31816000	3.72062900
C	-0.86403400	1.18866000	3.79879200

H	-1.84187400	-0.12453300	2.42847600
N	1.98132000	0.07904800	3.21795000
C	-1.23197500	2.87125000	-0.09511500
Si	0.09897100	2.01704400	-2.47446600
C	-1.69121000	-2.06985600	-0.95619900
Si	0.23503200	-1.05623300	-2.79905000
C	4.53885600	-0.91545800	-0.91311700
C	3.07940300	-2.41364400	0.34301000
H	0.26276100	-1.23037300	4.33341700
H	-1.50884600	-1.47070300	4.37995000
H	-0.53868400	-2.20527900	3.08095900
H	0.11813600	1.32208100	4.26907800
H	-1.61770900	1.05747100	4.58933100
H	-1.11499800	2.09805800	3.23607100
C	3.40884800	0.21290000	3.42164900
C	-2.42465500	2.31445900	0.68826900
C	-0.19153700	3.48835200	0.85181900
C	-1.76959500	3.97127200	-1.01929400
C	-1.13848500	2.37918700	-3.83716300
C	1.50946900	3.24627600	-2.51222300
O	0.80235500	0.52167200	-2.77276200
C	-2.03401800	-2.07038900	0.52940800
C	-2.94345700	-1.59267200	-1.71068700
C	-1.33294100	-3.49930500	-1.38111000
C	1.68583400	-2.20263000	-3.03708000
C	-0.89106300	-1.22826500	-4.29253100
C	4.89607200	0.43611700	-1.48560400
C	5.33726700	-2.01028200	-1.25394000
C	1.83106200	-2.73668200	1.12066600
C	3.91777900	-3.47580500	-0.01417900
C	3.65841700	1.30745800	4.45559000
C	4.00002700	-1.12262000	3.86812100
H	3.91210500	0.50951700	2.47723800
H	-3.18008500	1.92586800	-0.01071600
H	-2.88910400	3.08885800	1.31721100
H	-2.11065100	1.49575500	1.34825600
H	0.64246200	3.90946100	0.26984300
H	-0.63136300	4.29208200	1.46267100

H	0.22067000	2.72873200	1.53221700
H	-2.51759900	3.56576500	-1.71542000
H	-2.24724300	4.75876200	-0.41910900
H	-0.96475500	4.44692200	-1.60025600
H	-2.07773300	1.83105400	-3.67020700
H	-0.71447700	2.05518600	-4.80036000
H	-1.37473600	3.44986800	-3.91399900
H	1.18489100	4.28519500	-2.35720000
H	2.01247300	3.19025300	-3.48995400
H	2.24310600	2.99071500	-1.73249200
H	-1.15715200	-2.31434900	1.14182400
H	-2.82799400	-2.79971800	0.74281300
H	-2.41090800	-1.07900300	0.82490100
H	-3.12455200	-0.52928200	-1.49098300
H	-3.82633300	-2.17127900	-1.39869600
H	-2.83611900	-1.70663800	-2.79570000
H	-0.45275100	-3.85370700	-0.82453400
H	-2.17243000	-4.18431100	-1.19124100
H	-1.10285100	-3.55091600	-2.45662200
H	2.14654700	-2.02230700	-4.02031800
H	1.36247200	-3.25388000	-2.99990600
H	2.45147100	-2.05565500	-2.26340300
H	-1.29685000	-2.24198300	-4.42266000
H	-0.27113000	-1.00482500	-5.17564800
H	-1.72579100	-0.51479600	-4.27767500
H	4.07723100	0.82761400	-2.10942400
H	5.79590600	0.36266300	-2.10953200
H	5.07995900	1.17746800	-0.69449400
C	5.05357100	-3.29934700	-0.80338500
H	6.20533500	-1.84851400	-1.89841700
H	1.72874500	-3.82315500	1.24295500
H	0.94716400	-2.37080200	0.57872300
H	1.82867900	-2.27695000	2.11903400
H	3.65740300	-4.48210700	0.32458900
H	3.23343900	2.26177200	4.11333100
H	3.17647700	1.03883200	5.40763300
H	4.73516300	1.44517600	4.63156400
H	5.06976800	-1.01531600	4.09980500

H	3.47928500	-1.48138200	4.76880400
H	3.89735800	-1.88018400	3.07752500
C	5.94976600	-4.45864500	-1.14847200
H	6.75505900	-4.56157800	-0.40449500
H	6.42259400	-4.31805100	-2.12981800
H	5.39078900	-5.40384600	-1.16416500

Table S21

Ge=Sb-Prod

Atomic Number	Coordinates (Angstroms) X	Y	Z
Ge	0.01943600	0.26900200	-0.21159700
N	0.16072500	0.10977500	1.68927800
Sb	2.59077400	0.67589100	0.06161300
C	-0.77900200	-0.10345800	2.81155700
C	1.52259200	0.23042200	1.97686200
N	-0.74677200	1.85219000	-0.87756500
N	-0.64956500	-1.08052700	-1.32487100
C	3.69280000	-1.19935100	-0.17076700
C	-0.52639100	-1.40561500	3.58059100
C	-0.82209900	1.08499500	3.77653900
H	-1.77451000	-0.19144900	2.35936300
N	2.02048600	0.10403300	3.13319200
C	-1.33912600	2.89897800	-0.02020900
Si	-0.21339500	2.13824700	-2.53161400
C	-1.76645700	-2.00997700	-1.04441900
Si	0.07191400	-0.91495100	-2.92734500
C	4.99812400	-1.07087000	-0.69276100
C	3.22050400	-2.48216300	0.18955100
H	0.37941700	-1.32038000	4.19097600
H	-1.38440100	-1.61065400	4.23819000
H	-0.40529200	-2.26207500	2.90377800
H	0.14981300	1.20871800	4.26926000
H	-1.58690600	0.90468500	4.54662400
H	-1.07646600	2.01790900	3.25558300

C	3.44118600	0.23064800	3.36166900
C	-2.48000300	2.28765500	0.79874400
C	-0.26679600	3.51296200	0.89252000
C	-1.95425700	4.01766600	-0.87206500
C	-1.61061000	2.43640400	-3.74883600
C	1.11479500	3.44538700	-2.71590000
O	0.53574700	0.69430200	-2.93853200
C	-2.03700600	-2.05893200	0.45385400
C	-3.05817500	-1.53831800	-1.73165500
C	-1.39833400	-3.42133900	-1.52114600
C	1.60277900	-1.95823300	-3.14851800
C	-1.06493300	-1.17878400	-4.39786600
C	5.58058100	0.25898900	-1.11575300
C	5.80114600	-2.20900300	-0.83626100
C	1.83273800	-2.71528900	0.72438900
C	4.05840600	-3.58958600	0.03267500
C	3.68552900	1.24666000	4.47353100
C	4.03284600	-1.13484100	3.70484200
H	3.94925300	0.59992800	2.44204000
H	-3.25890800	1.90686400	0.12146300
H	-2.93134500	3.02872900	1.47573500
H	-2.11846500	1.45410000	1.41190600
H	0.52367700	3.97391500	0.27983600
H	-0.69227300	4.28612900	1.55109800
H	0.19691200	2.74274400	1.52628200
H	-2.74241100	3.62184800	-1.52799900
H	-2.40430300	4.77488200	-0.21439900
H	-1.20009900	4.52839300	-1.48905800
H	-2.48658300	1.81931300	-3.49701700
H	-1.26766500	2.15553800	-4.75691500
H	-1.92804600	3.48806200	-3.78037200
H	0.75628500	4.46749900	-2.52994300
H	1.51150400	3.40662700	-3.74214600
H	1.94485600	3.23751700	-2.02410000
H	-1.11996600	-2.28969900	1.01125600
H	-2.79571700	-2.81847900	0.68947800
H	-2.42801200	-1.08667200	0.79019400
H	-3.25688800	-0.48990400	-1.46138700

H	-3.91436300	-2.15241300	-1.41279000
H	-2.98652100	-1.60507300	-2.82394000
H	-0.49903000	-3.77822000	-0.99811900
H	-2.22161900	-4.12440600	-1.32614300
H	-1.19734900	-3.44149200	-2.60301700
H	2.05887400	-1.77196700	-4.13245600
H	1.35652800	-3.02913500	-3.08231600
H	2.35370400	-1.74041200	-2.37472500
H	-1.44466000	-2.20555500	-4.49603900
H	-0.46452000	-0.95933300	-5.29519300
H	-1.91831600	-0.48700300	-4.38928900
H	4.97044000	0.74229000	-1.89350900
H	6.59290500	0.12478300	-1.51753200
H	5.64180200	0.96238100	-0.27146600
C	5.35371900	-3.47728900	-0.47534800
H	6.80864200	-2.09543900	-1.24491500
H	1.68776400	-2.22114500	1.69690100
H	1.64509300	-3.78947800	0.85778100
H	1.07721300	-2.32247100	0.02683100
H	3.68120500	-4.57684300	0.31383700
H	3.25924100	2.22286400	4.20210700
H	3.20200800	0.90749400	5.40185300
H	4.76144100	1.37249200	4.66246900
H	5.10713400	-1.05025700	3.92496800
H	3.52452900	-1.55244500	4.58705200
H	3.90787100	-1.83333300	2.86415700
C	6.23011400	-4.69318100	-0.61884500
H	6.49430400	-5.10306900	0.36767000
H	7.16227800	-4.45394800	-1.14681700
H	5.71340500	-5.48835600	-1.17537600

Table S22

Ge=Bi-Prod

Atomic Number	Coordinates (Angstroms) X	Y	Z

Ge	0.01095300	0.28046500	-0.23986300
N	0.17201700	0.12179400	1.65857400
Bi	2.65256500	0.73106200	-0.05314700
C	-0.75999100	-0.12061300	2.78325000
C	1.52967300	0.27053000	1.94733900
N	-0.76383300	1.87589200	-0.87942500
N	-0.68883200	-1.05354800	-1.35711300
C	3.77750000	-1.25403700	-0.19331400
C	-0.48114500	-1.42596700	3.53713200
C	-0.82082900	1.05591100	3.76193700
H	-1.75564000	-0.22032500	2.33447000
N	2.03254000	0.14815300	3.10005800
C	-1.36147500	2.90547600	-0.00545600
Si	-0.28671900	2.16857200	-2.54774200
C	-1.78827000	-2.00099200	-1.06911400
Si	0.01832800	-0.87867000	-2.96422300
C	5.11861100	-1.14542000	-0.61206600
C	3.26376800	-2.52060100	0.15363100
H	0.43114400	-1.33411200	4.13692300
H	-1.32787000	-1.64786500	4.20389300
H	-0.35768200	-2.27495900	2.85150900
H	0.14760700	1.18643900	4.25949500
H	-1.58607500	0.85655800	4.52692000
H	-1.08539000	1.99172100	3.25138900
C	3.45132700	0.28495500	3.32964600
C	-2.49429800	2.27417700	0.80995200
C	-0.28938600	3.51585200	0.91032900
C	-1.98936600	4.03296700	-0.83636100
C	-1.71873200	2.46036900	-3.72549800
C	1.02427700	3.48923900	-2.77080700
O	0.46508500	0.73588100	-2.98564600
C	-2.03559200	-2.06598000	0.43216300
C	-3.09487300	-1.53965100	-1.73460500
C	-1.41058400	-3.40437800	-1.56240600
C	1.56024600	-1.90539600	-3.19073000
C	-1.12581700	-1.15520500	-4.42659000
C	5.74328300	0.17737500	-0.99498400
C	5.91846500	-2.29302400	-0.67513300

C	1.84296300	-2.71580800	0.61066800
C	4.09705600	-3.64151300	0.07751000
C	3.69087200	1.28613900	4.45578500
C	4.05566700	-1.08088800	3.64872500
H	3.95235800	0.67181500	2.41278100
H	-3.27286600	1.89739100	0.13002600
H	-2.94881800	3.00215500	1.49897700
H	-2.12451200	1.43452300	1.40948900
H	0.48832900	3.99999700	0.29882200
H	-0.71874000	4.27162600	1.58635600
H	0.18919300	2.74016600	1.52580000
H	-2.78455100	3.64397900	-1.48770300
H	-2.43380000	4.78045100	-0.16389800
H	-1.24375500	4.55356900	-1.45546300
H	-2.58196900	1.83385900	-3.45353500
H	-1.39942000	2.18629200	-4.74316600
H	-2.04665400	3.50905600	-3.74482800
H	0.66034900	4.50842000	-2.57956100
H	1.39445300	3.45038500	-3.80689100
H	1.87655000	3.29697300	-2.10178400
H	-1.10587500	-2.28683900	0.97275900
H	-2.77833000	-2.83935000	0.67374500
H	-2.43654200	-1.10250500	0.78119700
H	-3.30550700	-0.49793000	-1.44816200
H	-3.93834400	-2.17027800	-1.41399600
H	-3.03482700	-1.59094800	-2.82855000
H	-0.50036400	-3.75534500	-1.05471900
H	-2.22311900	-4.11810200	-1.36110800
H	-1.22504900	-3.41452700	-2.64707900
H	2.02906500	-1.69401300	-4.16350800
H	1.32008000	-2.97909400	-3.15265200
H	2.29935300	-1.70218200	-2.40116500
H	-1.49845500	-2.18473100	-4.52180300
H	-0.53395000	-0.93091000	-5.32837300
H	-1.98453800	-0.47000000	-4.41178600
H	5.19696600	0.66334100	-1.81769200
H	6.78129300	0.03614000	-1.32203400
H	5.75223200	0.88173200	-0.14845900

C	5.42743400	-3.55173200	-0.33472200
H	6.95812200	-2.19587200	-0.99967300
H	1.65824000	-2.19603600	1.56346500
H	1.62238400	-3.78230200	0.75658600
H	1.13453600	-2.31938600	-0.13311000
H	3.68967700	-4.61877300	0.35188100
H	3.25402800	2.26245900	4.20197100
H	3.21496600	0.92825600	5.38097000
H	4.76638300	1.41901800	4.64224000
H	5.13152200	-0.99228800	3.85978100
H	3.55865000	-1.51403100	4.52991800
H	3.92615400	-1.76777800	2.79912500
C	6.28955900	-4.78290100	-0.42987400
H	6.14600300	-5.43568400	0.44254900
H	7.35389400	-4.52105200	-0.49376500
H	6.03414900	-5.37049100	-1.32520900
